# Supplementary material for: Homeotic transformations and number changes in the vertebral column of Triturus newts
Source: PeerJ. 2015 Nov 10;3:e1397. doi: 10.7717/peerj.1397 (PMC4647568; doi:10.7717/peerj.1397)
Supplement: Table S1 — Overview of the analyzed samples of Triturus: species, geographic populations, their position relative to the species contact zone and number of specimens. # stands for ambiguous species allocation. Hybrid zones are generally narrow (Arntzen, Wielstra & Wallis, 2014). However, spatial and genetic data suggest that species displaced one another, and hybrid zones have been moving (Wielstra & Arntzen 2012; Wielstra et al., 2013), which—in the context of this study—widens them. Populations were assigned as “central” or “fringe” based upon their geographical position away (≥50 km) or close to (<50 km) congeneric species. [file peerj-03-1397-s001.docx]

Supplementary Table S1.

Overview of the analyzed samples of *Triturus*: species, geographic populations, their position relative to the species contact zone and number of specimens. # stands for ambiguous species allocation. Hybrid zones are generally narrow (**Arntzen**, Wielstra & Wallis, 2014). However, spatial and genetic data suggest that species displaced one another, and hybrid zones have been moving (Wielstra & Arntzen, 2012; Wielstra et al., 2013), which – in the context of this study – widens them. Populations were assigned as “central” or “fringe” based upon their geographical position away (>= 50km) or close to (< 50 km) congeneric species.

| Locality | Country | No | Latitude | Longitude | Position |
| --- | --- | --- | --- | --- | --- |
| ***T. pygmaeus*** |  |  |  |  |  |
| Chao das Pias | Portugal | 10 | 39 55 | -08 81 | central |
| Puerto de Galiz | Spain | 11 | 36 41 | -05 27 | central |
| Arichidona-Loja | Spain | 11 | 37 06 | -04 23 | central |
| Villalba | Spain | 12 | 40 38 | -04 00 | central |
| Rio Alberite | Spain | 1 | 36 24 | -05 39 | fringe |
| Venta del Charco | Spain | 10 | 38 12 | -04 16 | central |
| ***T. marmoratus*** |  |  |  |  |  |
| Confolens | France | 1 | 46 01 | 00 40 | central |
| Mayenne | France | 45 | 48 30 | -06 17 | central |
| El Berrueco | Spain | 8 | 40 54 | -03 53 | central |
| Rochechouart | France | 4 | 45 49 | 00 50 | central |
| ***T. ivanbureschi*** |  |  |  |  |  |
| Mersimbeleni | Turkey | 19 | 37 65 | 27 68 | central |
| Reşadiye | Turkey | 20 | 40 24 | 37 19 | central |
| Şerefiye | Turkey | 19 | 40 09 | 37 46 | central |
| Klaros | Turkey | 19 | 38 00 | 27 18 | central |
| Kalecik | Turkey | 15 | 40 08 | 33 35 | central |
| Arifiye | Turkey | 6 | 40 68 | 30 36 | central |
| Afyon | Turkey | 11 | 38 68 | 30 45 | central |
| Trabzon | Turkey | 4 | 40 99 | 39 73 | central |
| Tosya | Turkey | 18 | 41 01 | 34 03 | central |
| Bursa | Turkey | 20 | 40 11 | 29 03 | central |
| Guberevac | Serbia | 9 | 43 49 | 20 46 | fringe |
| Istanbul | Turkey | 9 | 41 02 | 28 57 | central |
| Karlovo | Bulgaria | 3 | 42 38 | 24 49 | central |
| Trešnja | Serbia | 34 | 44 36 | 20 35 | fringe |
| Dafnohori | Greece | 3 | 40 57 | 22 48 | fringe |
| Gornja Sabanta | Serbia | 1 | 43 54 | 21 00 | fringe |
| Karacaby | Turkey | 3 | 40 15 | 28 18 | central |
| Mitrašinci | FYR Macedonia | 12 | 41 75 | 22 76 | fringe |
| Resavica Pećina | Serbia | 3 | 44 05 | 21 60 | fringe |
| Sevlievo | Bulgaria | 2 | 43 46 | 25 21 | fringe |
| Sićevac | Serbia | 4 | 43 95 | 21 58 | fringe |
| Vitanovac | Serbia | 9 | 43 73 | 20 80 | fringe |
| Đurinci | Serbia | 2 | 44 49 | 20 65 | fringe |
| Kentriko | Greece | 13 | 41 16 | 22 90 | fringe |
| Levski | Bulgaria | 9 | 43 41 | 25 13 | fringe |
| Rakovski | Bulgaria | 4 | 42 26 | 24 96 | central |
| Bartin | Turkey | 9 | 41 37 | 32 20 | central |
| Adapazari | Turkey | 6 | 40 47 | 30 24 | central |
| Bigla | FYR Macedonia | 5 | 41 93 | 22 66 | fringe |
| Arandjelovac | Serbia | 8 | 44 28 | 20 66 | fringe |
| Grivac | Serbia | 9 | 43 96 | 20 66 | fringe |
| Vlasi # | Serbia | 31 | 42 98 | 22 63 | fringe |
| Berovo # | FYR Macedonia | 22 | 41 71 | 22 86 | fringe |
| ***T. karelinii*** |  |  |  |  |  |
| Ersi | Georgia | 6 | 41 90 | 45 47 | central |
| Tabasaranskii | Dagestan | 18 | 42 00 | 48 01 | central |
| Kutuzovsko lake | Ukraine | 15 | 44 75 | 30 24 | central |
| Dizabad | Iran | 2 | 49 18 | 34 18 | central |
| Akhaldaba | Georgia | 2 | 41 68 | 44 65 | central |
| ***T. mecedonicus*** |  |  |  |  |  |
| Rataje | Serbia | 55 | 43 29 | 21 08 | fringe |
| Galičica | FYR Macedonia | 15 | 41 04 | 20 52 | central |
| Todorovce | Serbia | 15 | 42 52 | 21 51 | central |
| Divčibare | Serbia | 31 | 44 11 | 19 93 | fringe |
| Višegrad | Bosnia-Hercegovina | 14 | 43 78 | 19 33 | central |
| Stanišinci | Serbia | 4 | 43 51 | 20 88 | fringe |
| Ano Kalliniki | Greece | 7 | 40 52 | 21 26 | central |
| Karan | Serbia | 6 | 43 88 | 19 92 | fringe |
| Manastir Tavna | Bosnia-Hercegovina | 7 | 44 60 | 19 06 | fringe |
| GornjaČadjavica | Bosnia-Hercegovina | 9 | 44 75 | 19 08 | fringe |
| Probistip | FYR Macedonia | 4 | 42 00 | 22 90 | central |
| Grčak | Serbia | 8 | 43 46 | 20 95 | fringe |
| Lučane | Serbia | 12 | 42 41 | 21 71 | central |
| Rtanj # | Serbia | 20 | 43 76 | 21 93 | fringe |
| Vranje # | Serbia | 19 | 42 60 | 21 85 | fringe |
| ***T. carnifex*** |  |  |  |  |  |
| Podstrmec | Slovenia | 19 | 45 48 | 14 34 | central |
| Bominaco | Italy | 5 | 42 35 | 13 40 | central |
| Farma | Italy | 6 | 43 13 | 11 16 | central |
| Haidlhof | Austria | 7 | 47 96 | 16 16 | fringe |
| Geneve | Switzerland | 38 | 46 25 | 06 30 | fringe |
| Sinac | Croatia | 2 | 44 49 | 15 22 | central |
| Etzmannsdorf | Austria | 1 | 48 65 | 15 75 | fringe |
| Fuscaldo | Italy | 12 | 39 41 | 16 03 | central |
| Locarno | Switzerland | 5 | 46 10 | 08 48 | central |
| Pisa | Italy | 3 | 43 71 | 10 40 | central |
| Firenze | Italy | 6 | 43 47 | 11 15 | central |
| Klein-Meiseldorf | Austria | 11 | 48 66 | 15 75 | fringe |
| Kramplje | Slovenia | 6 | 45 73 | 14 50 | central |
| Napoli | Italy | 2 | 40 85 | 14 25 | central |
| ***T. cristatus*** |  |  |  |  |  |
| BelaCrkva | Serbia | 33 | 44 88 | 21 54 | fringe |
| Miroč | Serbia | 34 | 44 48 | 22 33 | fringe |
| Negotin | Serbia | 20 | 44 25 | 22 53 | fringe |
| Mayenne | France | 88 | 48 30 | -06 17 | central |
| Lanckorona | Poland | 8 | 49 54 | 19 29 | central |
| Ambleteuse | France | 2 | 50 46 | 01 37 | central |
| Bor | Serbia | 4 | 44 03 | 22 13 | fringe |
| Sebis | Romania | 4 | 46 22 | 22 06 | central |
| Biel | Switzerland | 5 | 47 15 | 07 27 | central |
| Kladovo | Serbia | 2 | 44 55 | 22 55 | fringe |
| Klokočevac | Serbia | 4 | 44 33 | 22 20 | fringe |
| Ottenstein | Austria | 1 | 48 46 | 14 28 | fringe |
| Štubik | Serbia | 4 | 44 29 | 22 35 | fringe |
| Tirgovište | Romania | 9 | 44 93 | 25 45 | fringe |
| Videle | Romania | 10 | 44 26 | 25 51 | fringe |
| Canterbury | United Kingdom | 3 | 51 28 | 01 05 | central |
| Saint-Lô | France | 1 | 49 07 | -01 05 | fringe |
| Campeni | Romania | 10 | 46 38 | 23 08 | fringe |
| Limanowa | Poland | 5 | 49 43 | 20 25 | central |
| Milanovac | Serbia | 7 | 44 18 | 21 60 | fringe |
| Virfuri | Romania | 10 | 46 28 | 22 46 | fringe |
| Braila | Romania | 1 | 45 20 | 27 98 | central |
| Jabukovac | Serbia | 5 | 44 20 | 22 24 | fringe |
| Lukovo | Serbia | 1 | 43 48 | 21 50 | fringe |
| Peterborough | United Kingdom | 5 | 52 58 | -00 15 | central |
| Sinaia | Romania | 10 | 45 33 | 25 55 | fringe |
| ***T. dobrogicus*** |  |  |  |  |  |
| Ivanovo | Serbia | 51 | 44 73 | 20 70 | fringe |
| Opovo | Serbia | 20 | 45 03 | 20 44 | fringe |
| Kikinda | Serbia | 9 | 45 67 | 20 47 | central |
| Marchegg | Austria | 1 | 48 28 | 16 90 | fringe |
| Öcsöd | Hungary | 9 | 46 93 | 20 38 | central |
| Sebis | Romania | 5 | 46 36 | 22 10 | fringe |
| Svistov | Bulgaria | 2 | 43 61 | 25 35 | fringe |
| Szolnok | Hungary | 1 | 47 10 | 20 10 | central |
| Županja | Croatia | 6 | 45 08 | 18 70 | fringe |
| Debrc | Serbia | 26 | 44 60 | 19 86 | fringe |
| Podgorac | Croatia | 2 | 45 48 | 18 20 | central |
| Zimnicea | Romania | 2 | 43 65 | 25 35 | fringe |
| Tadten | Austria | 3 | 47 76 | 17 00 | fringe |
| Alap | Hungary | 7 | 46 80 | 18 68 | central |
| Beograd | Serbia | 6 | 44 83 | 20 50 | fringe |
| Drösing | Austria | 5 | 48 55 | 16 91 | fringe |
| Ečka | Serbia | 15 | 45 28 | 20 45 | central |
| Glušci | Serbia | 9 | 44 85 | 19 54 | fringe |
| Jamena | Serbia | 9 | 44 87 | 19 06 | fringe |
| Senta | Serbia | 15 | 45 91 | 20 10 | central |
| Donja Čađavica | Bosnia-Hercegovina | 11 | 44 75 | 19 10 | fringe |
| Dugo Selo | Croatia | 1 | 45 81 | 16 25 | fringe |
| Körmend | Hungary | 1 | 47 01 | 16 60 | fringe |

**References**

**Arntzen JW, Wielstra B, Wallis GP. 2014**. The modality of nine *Triturus* newt hybrid zones assessed with nuclear, mitochondrial and morphological data. *Biological Journal of the Linnean Society* **113**: 604-622 DOI: 10.1111/bij.12358.

**Wielstra B, Arntzen JW. 2012**. Postglacial species displacement in *Triturus* newts deduced from asymmetrically introgresed mitochondrial DNA and ecological niche models. *BMC Evolutionary Biology* **12**: 161 DOI:10.1186/1471-2148-12-161.

**Wielstra B, Crnobrnja-Isailović J, Litvinchuk SN, Reijnen BT, Skidmore AK, Sotiropoulis K, Toxopeus AG, Tzankov N, Vukov T, Arntzen JW.** **2013.** Tracing glacial refugia of *Triturus* newts based on mitochondrial DNA phylogeography and species distribution modeling. *Frontiers in Zoology* **10**:13 DOI:10.1186/1742-9994-10-13.
