# Supplementary material for: Homeotic transformations and number changes in the vertebral column of Triturus newts
Source: PeerJ. 2015 Nov 10;3:e1397. doi: 10.7717/peerj.1397 (PMC4647568; doi:10.7717/peerj.1397)
Supplement: Table S2 — Overview of the analyzed samples of Triturus: species, geographic populations, number of specimens, and variation in the number of vertebrae. For each species standard vertebral formula is given next to the species name. # stands for ambiguous species allocation. [file peerj-03-1397-s002.docx]

Supplementary Table S2.

Overview of the analyzed samples of *Triturus*: species, geographic populations, number of specimens, and variation in the number of vertebrae. For each species standard vertebral formula is given next to the species name. # stands for ambiguous species allocation.

| Locality | Country | No | 12 | 12.5 | 13 | 13.5 | 14 | 14.5 | 15 | 15.5 | 16 | 16.5 | 17 | 17.5 | 18 |
| --- | --- | --- | --- | --- | --- | --- | --- | --- | --- | --- | --- | --- | --- | --- | --- |
| ***T. pygmaeus***  **(1C 12T 1S)** |  |  |  |  |  |  |  |  |  |  |  |  |  |  |  |
| Chao das Pias | Portugal | 10 | 9 | 1 | 0 | 0 | 0 | 0 | 0 | 0 | 0 | 0 | 0 | 0 | 0 |
| Puerto de Galiz | Spain | 11 | 9 | 0 | 2 | 0 | 0 | 0 | 0 | 0 | 0 | 0 | 0 | 0 | 0 |
| Arichidona-Loja | Spain | 11 | 10 | 0 | 1 | 0 | 0 | 0 | 0 | 0 | 0 | 0 | 0 | 0 | 0 |
| Villalba | Spain | 12 | 12 | 0 | 0 | 0 | 0 | 0 | 0 | 0 | 0 | 0 | 0 | 0 | 0 |
| Rio Alberite | Spain | 1 | 1 | 0 | 0 | 0 | 0 | 0 | 0 | 0 | 0 | 0 | 0 | 0 | 0 |
| Venta del Charco | Spain | 10 | 10 | 0 | 0 | 0 | 0 | 0 | 0 | 0 | 0 | 0 | 0 | 0 | 0 |
| ***T. marmoratus***  **(1C 12T 1S)** |  |  |  |  |  |  |  |  |  |  |  |  |  |  |  |
| Confolens | France | 1 | 0 | 1 | 0 | 0 | 0 | 0 | 0 | 0 | 0 | 0 | 0 | 0 | 0 |
| Mayenne | France | 45 | 40 | 0 | 5 | 0 | 0 | 0 | 0 | 0 | 0 | 0 | 0 | 0 | 0 |
| El Berrueco | Spain | 8 | 4 | 2 | 2 | 0 | 0 | 0 | 0 | 0 | 0 | 0 | 0 | 0 | 0 |
| Rochechouart | France | 4 | 2 | 1 | 1 | 0 | 0 | 0 | 0 | 0 | 0 | 0 | 0 | 0 | 0 |
| ***T. ivanbureschi***  **(1C 13T 1S)** |  |  |  |  |  |  |  |  |  |  |  |  |  |  |  |
| Mersimbeleni | Turkey | 19 | 1 | 1 | 15 | 1 | 1 | 0 | 0 | 0 | 0 | 0 | 0 | 0 | 0 |
| Reşadiye | Turkey | 20 | 0 | 0 | 19 | 0 | 1 | 0 | 0 | 0 | 0 | 0 | 0 | 0 | 0 |
| Şerefiye | Turkey | 19 | 0 | 0 | 16 | 1 | 2 | 0 | 0 | 0 | 0 | 0 | 0 | 0 | 0 |
| Klaros | Turkey | 19 | 0 | 2 | 17 | 0 | 0 | 0 | 0 | 0 | 0 | 0 | 0 | 0 | 0 |
| Kalecik | Turkey | 15 | 0 | 0 | 12 | 1 | 2 | 0 | 0 | 0 | 0 | 0 | 0 | 0 | 0 |
| Arifiye | Turkey | 6 | 0 | 0 | 4 | 0 | 2 | 0 | 0 | 0 | 0 | 0 | 0 | 0 | 0 |
| Afyon | Turkey | 11 | 0 | 0 | 8 | 1 | 2 | 0 | 0 | 0 | 0 | 0 | 0 | 0 | 0 |
| Trabzon | Turkey | 4 | 0 | 0 | 4 | 0 | 0 | 0 | 0 | 0 | 0 | 0 | 0 | 0 | 0 |
| Tosya | Turkey | 18 | 0 | 0 | 17 | 0 | 1 | 0 | 0 | 0 | 0 | 0 | 0 | 0 | 0 |
| Bursa | Turkey | 20 | 0 | 0 | 20 | 0 | 0 | 0 | 0 | 0 | 0 | 0 | 0 | 0 | 0 |
| Guberevac | Serbia | 9 | 0 | 0 | 8 | 0 | 1 | 0 | 0 | 0 | 0 | 0 | 0 | 0 | 0 |
| Istanbul | Turkey | 9 | 0 | 0 | 6 | 0 | 3 | 0 | 0 | 0 | 0 | 0 | 0 | 0 | 0 |
| Karlovo | Bulgaria | 3 | 0 | 0 | 2 | 0 | 1 | 0 | 0 | 0 | 0 | 0 | 0 | 0 | 0 |
| Trešnja | Serbia | 34 | 0 | 0 | 16 | 1 | 17 | 0 | 0 | 0 | 0 | 0 | 0 | 0 | 0 |
| Dafnohori | Greece | 3 | 0 | 0 | 1 | 0 | 2 | 0 | 0 | 0 | 0 | 0 | 0 | 0 | 0 |
| Gornja Sabanta | Serbia | 1 | 0 | 0 | 1 | 0 | 0 | 0 | 0 | 0 | 0 | 0 | 0 | 0 | 0 |
| Karacaby | Turkey | 3 | 0 | 0 | 2 | 0 | 1 | 0 | 0 | 0 | 0 | 0 | 0 | 0 | 0 |
| Mitrašinci | FYR Macedonia | 12 | 1 | 0 | 8 | 0 | 3 | 0 | 0 | 0 | 0 | 0 | 0 | 0 | 0 |
| Resavica Pećina | Serbia | 3 | 0 | 0 | 1 | 1 | 1 | 0 | 0 | 0 | 0 | 0 | 0 | 0 | 0 |
| Sevlievo | Bulgaria | 2 | 0 | 0 | 2 | 0 | 0 | 0 | 0 | 0 | 0 | 0 | 0 | 0 | 0 |
| Sićevac | Serbia | 4 | 0 | 0 | 3 | 0 | 1 | 0 | 0 | 0 | 0 | 0 | 0 | 0 | 0 |
| Vitanovac | Serbia | 9 | 0 | 0 | 5 | 1 | 2 | 1 | 0 | 0 | 0 | 0 | 0 | 0 | 0 |
| Đurinci | Serbia | 2 | 0 | 0 | 0 | 0 | 2 | 0 | 0 | 0 | 0 | 0 | 0 | 0 | 0 |
| Kentriko | Greece | 13 | 0 | 0 | 8 | 0 | 5 | 0 | 0 | 0 | 0 | 0 | 0 | 0 | 0 |
| Levski | Bulgaria | 9 | 0 | 0 | 7 | 1 | 1 | 0 | 0 | 0 | 0 | 0 | 0 | 0 | 0 |
| Rakovski | Bulgaria | 4 | 0 | 0 | 2 | 1 | 1 | 0 | 0 | 0 | 0 | 0 | 0 | 0 | 0 |
| Bartin | Turkey | 9 | 0 | 0 | 8 | 0 | 1 | 0 | 0 | 0 | 0 | 0 | 0 | 0 | 0 |
| Adapazari | Turkey | 6 | 0 | 0 | 6 | 0 | 0 | 0 | 0 | 0 | 0 | 0 | 0 | 0 | 0 |
| Bigla | FYR Macedonia | 5 | 0 | 0 | 5 | 0 | 0 | 0 | 0 | 0 | 0 | 0 | 0 | 0 | 0 |
| Aranđelovac | Serbia | 8 | 0 | 0 | 8 | 0 | 0 | 0 | 0 | 0 | 0 | 0 | 0 | 0 | 0 |
| Grivac | Serbia | 9 | 1 | 0 | 8 | 0 | 0 | 0 | 0 | 0 | 0 | 0 | 0 | 0 | 0 |
| Vlasi # | Serbia | 31 | 0 | 0 | 11 | 3 | 16 | 0 | 1 | 0 | 0 | 0 | 0 | 0 | 0 |
| Berovo # | FYR Macedonia | 22 | 0 | 0 | 10 | 2 | 9 | 0 | 1 | 0 | 0 | 0 | 0 | 0 | 0 |
| ***T. karelinii***  **(1C 13T 1S)** |  |  |  |  |  |  |  |  |  |  |  |  |  |  |  |
| Ersi | Georgia | 6 | 0 | 0 | 6 | 0 | 0 | 0 | 0 | 0 | 0 | 0 | 0 | 0 | 0 |
| Tabasaranskii | Dagestan | 18 | 0 | 1 | 17 | 0 | 0 | 0 | 0 | 0 | 0 | 0 | 0 | 0 | 0 |
| Kutuzovsko lake | Ukraine | 15 | 0 | 0 | 15 | 0 | 0 | 0 | 0 | 0 | 0 | 0 | 0 | 0 | 0 |
| Dizabad | Iran | 2 | 0 | 0 | 1 | 0 | 1 | 0 | 0 | 0 | 0 | 0 | 0 | 0 | 0 |
| Akhaldaba | Georgia | 2 | 0 | 0 | 1 | 0 | 1 | 0 | 0 | 0 | 0 | 0 | 0 | 0 | 0 |
| ***T. mecedonicus***  **(1C 14T 1S)** |  |  |  |  |  |  |  |  |  |  |  |  |  |  |  |
| Rataje | Serbia | 55 | 0 | 0 | 5 | 1 | 48 | 1 | 0 | 0 | 0 | 0 | 0 | 0 | 0 |
| Galičica | FYR Macedonia | 15 | 0 | 0 | 0 | 0 | 14 | 1 | 0 | 0 | 0 | 0 | 0 | 0 | 0 |
| Todorovce | Serbia | 15 | 0 | 0 | 0 | 1 | 14 | 0 | 0 | 0 | 0 | 0 | 0 | 0 | 0 |
| Divčibare | Serbia | 31 | 0 | 0 | 5 | 0 | 26 | 0 | 0 | 0 | 0 | 0 | 0 | 0 | 0 |
| Višegrad | Bosnia-Hercegovina | 14 | 0 | 0 | 2 | 2 | 9 | 0 | 1 | 0 | 0 | 0 | 0 | 0 | 0 |
| Stanišinci | Serbia | 4 | 0 | 0 | 0 | 0 | 4 | 0 | 0 | 0 | 0 | 0 | 0 | 0 | 0 |
| Ano Kalliniki | Greece | 7 | 0 | 0 | 0 | 1 | 6 | 0 | 0 | 0 | 0 | 0 | 0 | 0 | 0 |
| Karan | Serbia | 6 | 0 | 0 | 0 | 0 | 6 | 0 | 0 | 0 | 0 | 0 | 0 | 0 | 0 |
| Manastir Tavna | Bosnia-Hercegovina | 7 | 0 | 0 | 1 | 0 | 5 | 0 | 1 | 0 | 0 | 0 | 0 | 0 | 0 |
| Gornja Čađavica | Bosnia-Hercegovina | 9 | 0 | 0 | 0 | 0 | 4 | 1 | 2 | 0 | 2 | 0 | 0 | 0 | 0 |
| Probistip | Macedonia | 4 | 0 | 0 | 4 | 0 | 2 | 0 | 0 | 0 | 0 | 0 | 0 | 0 | 0 |
| Grčak | Serbia | 8 | 0 | 0 | 4 | 0 | 4 | 0 | 0 | 0 | 0 | 0 | 0 | 0 | 0 |
| Lučane | Serbia | 12 | 0 | 0 | 3 | 1 | 8 | 0 | 0 | 0 | 0 | 0 | 0 | 0 | 0 |
| Rtanj # | Serbia | 20 | 0 | 0 | 6 | 1 | 11 | 1 | 1 | 0 | 0 | 0 | 0 | 0 | 0 |
| Vranje # | Serbia | 19 | 0 | 0 | 3 | 0 | 14 | 1 | 1 | 0 | 0 | 0 | 0 | 0 | 0 |
| ***T. carnifex***  **(1C 14T 1S)** |  |  |  |  |  |  |  |  |  |  |  |  |  |  |  |
| Podstrmec | Slovenia | 19 | 0 | 0 | 1 | 1 | 17 | 0 | 0 | 0 | 0 | 0 | 0 | 0 | 0 |
| Bominaco | Italy | 5 | 0 | 0 | 0 | 0 | 2 | 0 | 3 | 0 | 0 | 0 | 0 | 0 | 0 |
| Farma | Italy | 6 | 0 | 0 | 0 | 0 | 6 | 0 | 0 | 0 | 0 | 0 | 0 | 0 | 0 |
| Haidlhof | Austria | 7 | 0 | 0 | 0 | 0 | 4 | 0 | 3 | 0 | 0 | 0 | 0 | 0 | 0 |
| Geneve | Switzerland | 38 | 0 | 0 | 4 | 0 | 31 | 0 | 3 | 0 | 0 | 0 | 0 | 0 | 0 |
| Sinac | Croatia | 2 | 0 | 0 | 0 | 1 | 1 | 0 | 0 | 0 | 0 | 0 | 0 | 0 | 0 |
| Etzmannsdorf | Austria | 1 | 0 | 0 | 0 | 0 | 1 | 0 | 0 | 0 | 0 | 0 | 0 | 0 | 0 |
| Fuscaldo | Italy | 12 | 0 | 0 | 1 | 0 | 10 | 0 | 0 | 0 | 1 | 0 | 0 | 0 | 0 |
| Locarno | Switzerland | 5 | 0 | 0 | 1 | 0 | 4 | 0 | 0 | 0 | 0 | 0 | 0 | 0 | 0 |
| Pisa | Italy | 3 | 0 | 0 | 0 | 0 | 3 | 0 | 0 | 0 | 0 | 0 | 0 | 0 | 0 |
| Firenze | Italy | 6 | 0 | 0 | 0 | 2 | 4 | 0 | 0 | 0 | 0 | 0 | 0 | 0 | 0 |
| Klein-Meiseldorf | Austria | 11 | 0 | 0 | 0 | 0 | 1 | 3 | 7 | 0 | 0 | 0 | 0 | 0 | 0 |
| Kramplje | Slovenia | 6 | 0 | 0 | 0 | 0 | 6 | 0 | 0 | 0 | 0 | 0 | 0 | 0 | 0 |
| Napoli | Italy | 2 | 0 | 0 | 1 | 1 | 0 | 0 | 0 | 0 | 0 | 0 | 0 | 0 | 0 |
| ***T. cristatus***  **(1C 15T 1S)** |  |  |  |  |  |  |  |  |  |  |  |  |  |  |  |
| Bela Crkva | Serbia | 33 | 0 | 0 | 0 | 0 | 0 | 0 | 20 | 3 | 10 | 0 | 0 | 0 | 0 |
| Miroč | Serbia | 34 | 0 | 0 | 0 | 0 | 4 | 0 | 30 | 0 | 0 | 0 | 0 | 0 | 0 |
| Negotin | Serbia | 20 | 0 | 0 | 0 | 0 | 1 | 0 | 17 | 0 | 2 | 0 | 0 | 0 | 0 |
| Mayenne | France | 88 | 0 | 0 | 1 | 0 | 4 | 1 | 71 | 4 | 7 | 0 | 0 | 0 | 0 |
| Lanckorona | Poland | 8 | 0 | 0 | 0 | 0 | 1 | 0 | 7 | 0 | 0 | 0 | 0 | 0 | 0 |
| Ambleteuse | France | 2 | 0 | 0 | 0 | 0 | 0 | 0 | 1 | 1 | 0 | 0 | 0 | 0 | 0 |
| Bor | Serbia | 4 | 0 | 0 | 0 | 0 | 0 | 0 | 4 | 0 | 0 | 0 | 0 | 0 | 0 |
| Sebis | Romania | 4 | 0 | 0 | 0 | 0 | 0 | 0 | 4 | 0 | 0 | 0 | 0 | 0 | 0 |
| Biel | Switzerland | 5 | 0 | 0 | 0 | 0 | 0 | 0 | 3 | 0 | 2 | 0 | 0 | 0 | 0 |
| Kladovo | Serbia | 2 | 0 | 0 | 0 | 0 | 0 | 0 | 0 | 0 | 1 | 0 | 1 | 0 | 0 |
| Klokočevac | Serbia | 4 | 0 | 0 | 0 | 0 | 2 | 0 | 2 | 0 | 0 | 0 | 0 | 0 | 0 |
| Ottenstein | Austria | 1 | 0 | 0 | 0 | 0 | 0 | 0 | 1 | 0 | 0 | 0 | 0 | 0 | 0 |
| Štubik | Serbia | 4 | 0 | 0 | 0 | 0 | 0 | 0 | 4 | 0 | 0 | 0 | 0 | 0 | 0 |
| Tirgovište | Romania | 9 | 0 | 0 | 0 | 0 | 0 | 1 | 8 | 0 | 0 | 0 | 0 | 0 | 0 |
| Videle | Romania | 10 | 0 | 0 | 0 | 0 | 2 | 0 | 8 | 0 | 0 | 0 | 0 | 0 | 0 |
| Canterbury | United Kingdom | 3 | 0 | 0 | 0 | 0 | 1 | 0 | 2 | 0 | 0 | 0 | 0 | 0 | 0 |
| Saint-Lô | France | 1 | 0 | 0 | 0 | 0 | 0 | 0 | 1 | 0 | 0 | 0 | 0 | 0 | 0 |
| Campeni | Romania | 10 | 0 | 0 | 0 | 0 | 0 | 1 | 9 | 0 | 0 | 0 | 0 | 0 | 0 |
| Limanowa | Poland | 5 | 0 | 0 | 0 | 0 | 0 | 0 | 5 | 0 | 0 | 0 | 0 | 0 | 0 |
| Milanovac | Serbia | 7 | 0 | 0 | 0 | 0 | 1 | 1 | 5 | 0 | 0 | 0 | 0 | 0 | 0 |
| Virfuri | Romania | 10 | 0 | 0 | 0 | 0 | 0 | 0 | 9 | 0 | 0 | 0 | 1 | 0 | 0 |
| Braila | Romania | 1 | 0 | 0 | 0 | 0 | 0 | 0 | 0 | 0 | 1 | 0 | 0 | 0 | 0 |
| Jabukovac | Serbia | 5 | 0 | 0 | 0 | 0 | 0 | 0 | 5 | 0 | 0 | 0 | 0 | 0 | 0 |
| Lukovo | Serbia | 1 | 0 | 0 | 1 | 0 | 0 | 0 | 0 | 0 | 0 | 0 | 0 | 0 | 0 |
| Peterborough | United Kingdom | 5 | 0 | 0 | 0 | 0 | 0 | 0 | 4 | 0 | 1 | 0 | 0 | 0 | 0 |
| Sinaia | Romania | 10 | 0 | 0 | 0 | 0 | 1 | 1 | 8 | 0 | 0 | 0 | 0 | 0 | 0 |
| ***T. dobrogicus***  **(1C 17T 1S)** |  |  |  |  |  |  |  |  |  |  |  |  |  |  |  |
| Ivanovo | Serbia | 51 | 0 | 0 | 0 | 0 | 0 | 0 | 0 | 0 | 19 | 3 | 27 | 1 | 1 |
| Opovo | Serbia | 20 | 0 | 0 | 0 | 0 | 0 | 0 | 1 | 0 | 7 | 3 | 9 | 0 | 0 |
| Kikinda | Serbia | 9 | 0 | 0 | 0 | 0 | 0 | 0 | 0 | 0 | 1 | 0 | 8 | 0 | 0 |
| Marchegg | Austria | 1 | 0 | 0 | 0 | 0 | 0 | 0 | 0 | 0 | 0 | 0 | 1 | 0 | 0 |
| Öcsöd | Hungary | 9 | 0 | 0 | 0 | 0 | 0 | 0 | 0 | 0 | 0 | 0 | 8 | 0 | 1 |
| Sebis | Romania | 5 | 0 | 0 | 0 | 0 | 0 | 0 | 2 | 0 | 1 | 0 | 2 | 0 | 0 |
| Svistov | Bulgaria | 2 | 0 | 0 | 0 | 0 | 0 | 0 | 0 | 0 | 0 | 0 | 2 | 0 | 0 |
| Szolnok | Hungary | 1 | 0 | 0 | 0 | 0 | 0 | 0 | 0 | 0 | 0 | 0 | 1 | 0 | 0 |
| Županja | Croatia | 6 | 0 | 0 | 0 | 0 | 0 | 0 | 0 | 0 | 6 | 0 | 0 | 0 | 0 |
| Debrc | Serbia | 26 | 0 | 0 | 0 | 0 | 0 | 0 | 0 | 0 | 15 | 3 | 8 | 0 | 0 |
| Podgorac | Croatia | 2 | 0 | 0 | 0 | 0 | 0 | 0 | 0 | 0 | 1 | 0 | 1 | 0 | 0 |
| Zimnicea | Romania | 2 | 0 | 0 | 0 | 0 | 0 | 0 | 0 | 0 | 2 | 0 | 0 | 0 | 0 |
| Tadten | Austria | 3 | 0 | 0 | 0 | 0 | 0 | 0 | 0 | 0 | 3 | 0 | 0 | 0 | 0 |
| Alap | Hungary | 7 | 0 | 0 | 0 | 0 | 0 | 0 | 1 | 0 | 5 | 0 | 1 | 0 | 0 |
| Belgrad | Serbia | 6 | 0 | 0 | 0 | 0 | 0 | 0 | 0 | 0 | 2 | 0 | 4 | 0 | 0 |
| Drösing | Austria | 5 | 0 | 0 | 0 | 0 | 0 | 0 | 0 | 0 | 0 | 0 | 5 | 0 | 0 |
| Ečka | Serbia | 15 | 0 | 0 | 0 | 0 | 0 | 0 | 1 | 0 | 3 | 1 | 10 | 0 | 0 |
| Glušci | Serbia | 9 | 0 | 0 | 0 | 0 | 0 | 0 | 0 | 0 | 5 | 0 | 4 | 0 | 0 |
| Jamena | Serbia | 9 | 0 | 0 | 0 | 0 | 0 | 0 | 0 | 0 | 6 | 1 | 2 | 0 | 0 |
| Senta | Serbia | 15 | 0 | 0 | 0 | 0 | 0 | 0 | 0 | 0 | 1 | 0 | 14 | 0 | 0 |
| Donja Čađavica | Bosnia-Hercegovina | 11 | 0 | 0 | 0 | 0 | 1 | 1 | 6 | 0 | 3 | 0 | 0 | 0 | 0 |
| Dugo Selo | Croatia | 1 | 0 | 0 | 0 | 0 | 0 | 0 | 0 | 0 | 0 | 0 | 1 | 0 | 0 |
| Körmend | Hungary | 1 | 0 | 0 | 0 | 0 | 0 | 0 | 0 | 0 | 0 | 0 | 1 | 0 | 0 |
