# Supplementary material for: Homeotic transformations and number changes in the vertebral column of Triturus newts
Source: PeerJ. 2015 Nov 10;3:e1397. doi: 10.7717/peerj.1397 (PMC4647568; doi:10.7717/peerj.1397)
Supplement: Table S3 — We determined the vertebral formula by counting the number of cervical (C), thoracic (T) and sacral vertebrae (S). The caudosacral and caudal regions (Cd) are excluded from our formula. Homeotic transformations of thoracic vertebra into sacral vertebra, or vice versa (transitional sacral vertebra having half of the identity of thoracic vertebra and half of the identity of sacral vertebra) were assigned 0.5 and this score was added to the number of complete thoracic vertebrae. Only complete changes of identity on one side of the vertebrae (on one side thoracic and on one side sacral) were declared transitional and scored. Collection abbreviations: IBISS, Institute for Biological Research “Siniša Stanković” Belgrade; ZMA.RenA, Naturalis Biodiversity Center, Leiden. [file peerj-03-1397-s003.pdf]

The vertebral formulae of 1436 adult newts that originate from 126 populations of all eight species of *Triturus* newts. We determined the vertebral formula by counting the number of cervical (C), thoracic (T) and sacral vertebrae (S). The caudosacral and caudal regions (Cd) are excluded from our formula. Homeotic transformations of thoracic vertebra into sacral vertebra, or vice versa (transitional sacral vertebra having half of the identity of thoracic vertebra and half of the identity of sacral vertebra) were assigned 0.5 and this score was added to the number of complete thoracic vertebrae. Only complete changes of identity on one side of the vertebrae (on one side thoracic and on one side sacral) were declared transitional and scored. Collection abbreviations: IBISS - Institute for Biological Research "Siniša Stanković" Belgrade; ZMA.RenA - Naturalis Biodiversity Center, Leiden.

Species	Population	Collection_no	Vertebral formula	Score
T. pygmaeus	Chao das Pias	IBISS_G22582	1C 11T 1S_Cd	12
T. pygmaeus	Chao das Pias	IBISS_G22579	1C 11T 0.5T/S 0.5S/Cd_Cd	12.5
T. pygmaeus	Chao das Pias	IBISS_G22576	1C 11T 1S_Cd	12
T. pygmaeus	Chao das Pias	IBISS_G22577	1C 11T 1S_Cd	12
T. pygmaeus	Chao das Pias	IBISS_G22584	1C 11T 1S_Cd	12
T. pygmaeus	Chao das Pias	IBISS_G22578	1C 11T 1S_Cd	12
T. pygmaeus	Chao das Pias	IBISS_G22581	1C 11T 1S_Cd	12
T. pygmaeus	Chao das Pias	IBISS_G22585	1C 11T 1S_Cd	12
T. pygmaeus	Chao das Pias	IBISS_G22583	1C 11T 1S_Cd	12
T. pygmaeus	Chao das Pias	IBISS_G22575	1C 11T 1S_Cd	12
T. pygmaeus	Puerto de Galiz	ZMA.RenA_9148	1C 11T 1S_Cd	12
T. pygmaeus	Puerto de Galiz	ZMA.RenA_9148	1C 11T 1S_Cd	12
T. pygmaeus	Arichidona-Loja	ZMA.RenA_9087	1C 11T 1S_Cd	12
T. pygmaeus	Arichidona-Loja	ZMA.RenA_9087	1C 11T 1S_Cd	12
T. pygmaeus	Arichidona-Loja	ZMA.RenA_9087	1C 11T 1S_Cd	12
T. pygmaeus	Arichidona-Loja	ZMA.RenA_9087	1C 11T 1S_Cd	12
T. pygmaeus	Arichidona-Loja	ZMA.RenA_9087	1C 11T 1S_Cd	12
T. pygmaeus	Arichidona-Loja	ZMA.RenA_9087	1C 12T 1S_Cd	13
T. pygmaeus	Arichidona-Loja	ZMA.RenA_9087	1C 11T 1S_Cd	12
T. pygmaeus	Arichidona-Loja	ZMA.RenA_9087	1C 11T 1S_Cd	12
T. pygmaeus	Arichidona-Loja	ZMA.RenA_9087	1C 11T 1S_Cd	12
T. pygmaeus	Arichidona-Loja	ZMA.RenA_9087	1C 11T 1S_Cd	12
T. pygmaeus	Arichidona-Loja	ZMA.RenA_9087	1C 11T 1S_Cd	12
T. pygmaeus	Villalba	ZMA.RenA_7615	1C 11T 1S_Cd	12
T. pygmaeus	Villalba	ZMA.RenA_7615	1C 11T 1S_Cd	12
T. pygmaeus	Villalba	ZMA.RenA_7615	1C 11T 1S_Cd	12

T. pygmaeus	Villalba	ZMA.RenA_7615	1C 11T 1S_Cd	12
T. pygmaeus	Villalba	ZMA.RenA_7615	1C 11T 1S_Cd	12
T. pygmaeus	Villalba	ZMA.RenA_7615	1C 11T 1S_Cd	12
T. pygmaeus	Villalba	ZMA.RenA_7615	1C 12T 1S_Cd	12
T. pygmaeus	Villalba	ZMA.RenA_7615	1C 11T 1S_Cd	12
T. pygmaeus	Villalba	ZMA.RenA_7615	1C 11T 1S_Cd	12
T. pygmaeus	Villalba	ZMA.RenA_7615	1C 11T 1S_Cd	12
T. pygmaeus	Villalba	ZMA.RenA_7615	1C 11T 1S_Cd	12
T. pygmaeus	Villalba	ZMA.RenA_7615	1C 11T 1S_Cd	12
T. pygmaeus	Rio Alberite	ZMA.RenA_7618	1C 11T 1S_Cd	12
T. pygmaeus	Puerto de Galiz	ZMA.RenA_ 7676	1C 11T 1S_Cd	12
T. pygmaeus	Puerto de Galiz	ZMA.RenA_ 7676	1C 12T 1S_Cd	13
T. pygmaeus	Puerto de Galiz	ZMA.RenA_7676	1C 11T 1S_Cd	12
T. pygmaeus	Puerto de Galiz	ZMA.RenA_ 7676	1C 11T 1S_Cd	12
T. pygmaeus	Puerto de Galiz	ZMA.RenA_ 7676	1C 11T 1S_Cd	12
T. pygmaeus	Puerto de Galiz	ZMA.RenA_ 7676	1C 11T 1S_Cd	11
T. pygmaeus	Puerto de Galiz	ZMA.RenA_ 7676	1C 11T 1S_Cd	12
T. pygmaeus	Puerto de Galiz	ZMA.RenA_ 7676	1C 11T 1S_Cd	12
T. pygmaeus	Puerto de Galiz	ZMA.RenA_ 7676	1C 11T 1S_Cd	12
T. pygmaeus	Venta del Charco	ZMA.RenA_ 7677	1C 11T 1S_Cd	12
T. pygmaeus	Venta del Charco	ZMA.RenA_ 7677	1C 11T 1S_Cd	12
T. pygmaeus	Venta del Charco	ZMA.RenA_7677	1C 11T 1S_Cd	12
T. pygmaeus	Venta del Charco	ZMA.RenA_ 7677	1C 11T 1S_Cd	12
T. pygmaeus	Venta del Charco	ZMA.RenA_ 7677	1C 11T 1S_Cd	12
T. pygmaeus	Venta del Charco	ZMA.RenA_ 7677	1C 11T 1S_Cd	12
T. pygmaeus	Venta del Charco	ZMA.RenA_ 7677	1C 11T 1S_Cd	12
T. pygmaeus	Venta del Charco	ZMA.RenA_7677	1C 11T 1S_Cd	12
T. pygmaeus	Venta del Charco	ZMA.RenA_7677	1C 11T 1S_Cd	12
T. pygmaeus	Venta del Charco	ZMA.RenA_ 7677	1C 11T 1S_Cd	12
T. marmoratus	Confolens	ZMA.RenA_9095	1C 11T 0.5T/S 0.5S/Cd_Cd	12.5
T. marmoratus	Mayenne	ZMA.RenA_9267	1C 11T 1S_Cd	12
T. marmoratus	Mayenne	ZMA.RenA_9267	1C 11T 1S_Cd	12
T. marmoratus	Mayenne	ZMA.RenA_9267	1C 11T 1S_Cd	12
T. marmoratus	Mayenne	ZMA.RenA_7418	0.5C/T 11.5T 1S_Cd	12

T. marmoratus	Mayenne	ZMA.RenA_7418	1C 11T 1S_Cd	12
T. marmoratus	Mayenne	ZMA.RenA_7418	1C 11T 1S_Cd	12
T. marmoratus	Mayenne	ZMA.RenA_7418	1C 11T 1S_Cd	12
T. marmoratus	Mayenne	ZMA.RenA_7418	1C 11T 1S_Cd	12
T. marmoratus	Mayenne	ZMA.RenA_7418	1C 11T 1S_Cd	12
T. marmoratus	Mayenne	ZMA.RenA_7551	1C 11T 1S_Cd	12
T. marmoratus	Mayenne	ZMA.RenA_7551	1C 11T 1S_Cd	12
T. marmoratus	Mayenne	ZMA.RenA_7551	1C 11T 1S_Cd	12
T. marmoratus	Mayenne	ZMA.RenA_7551	1C 12T 1S_Cd	13
T. marmoratus	Mayenne	ZMA.RenA_7551	1C 11T 1S_Cd	12
T. marmoratus	Mayenne	ZMA.RenA_7551	1C 12T 1S_Cd	13
T. marmoratus	Mayenne	ZMA.RenA_7551	1C 11T 1S_Cd	12
T. marmoratus	El Berrueco	ZMA.RenA_7614	1C 12T 1S_Cd	13
T. marmoratus	El Berrueco	ZMA.RenA_7614	1C 11T 0.5T/S 0.5S/Cd_Cd	12.5
T. marmoratus	El Berrueco	ZMA.RenA_7614	1C 11T 1S_Cd	12
T. marmoratus	El Berrueco	ZMA.RenA_7614	1C 11T 1S_Cd	12
T. marmoratus	El Berrueco	ZMA.RenA_7614	1C 11T 1S_Cd	12
T. marmoratus	El Berrueco	ZMA.RenA_7614	1C 11T 1S_Cd	12
T. marmoratus	El Berrueco	ZMA.RenA_7614	1C 11T 0.5T/S 0.5S/Cd_Cd	12.5
T. marmoratus	El Berrueco	ZMA.RenA_7614	1C 12T 1S_Cd	13
T. marmoratus	Mayenne	ZMA.RenA_8049	1C 11T 1S_Cd	12
T. marmoratus	Mayenne	ZMA.RenA_9074	1C 11T 1S_Cd	12
T. marmoratus	Mayenne	ZMA.RenA_9074	1C 11T 1S_Cd	12
T. marmoratus	Mayenne	ZMA.RenA_9074	1C 11T 1S_Cd	12
T. marmoratus	Mayenne	ZMA.RenA_9074	1C 11T 1S_Cd	12
T. marmoratus	Mayenne	ZMA.RenA_9074	1C 12T 1S_Cd	13
T. marmoratus	Mayenne	ZMA.RenA_9074	1C 11T 1S_Cd	12
T. marmoratus	Mayenne	ZMA.RenA_9074	1C 11T 1S_Cd	12
T. marmoratus	Mayenne	ZMA.RenA_9074	1C 11T 1S_Cd	12
T. marmoratus	Mayenne	ZMA.RenA_9074	1C 11T 1S_Cd	12
T. marmoratus	Mayenne	ZMA.RenA_9074	1C 11T 1S_Cd	12
T. marmoratus	Mayenne	ZMA.RenA_9074	1C 11T 1S_Cd	12
T. marmoratus	Mayenne	ZMA.RenA_9075	1C 11T 1S_Cd	12
T. marmoratus	Mayenne	ZMA.RenA_9076	1C 11T 1S_Cd	12

T. marmoratus	Mayenne	ZMA.RenA_9076	1C 11T 1S_Cd	12
T. marmoratus	Mayenne	ZMA.RenA_9076	1C 11T 1S_Cd	12
T. marmoratus	Mayenne	ZMA.RenA_9077	1C 11T 1S_Cd	12
T. marmoratus	Mayenne	ZMA.RenA_9077	1C 11T 1S_Cd	12
T. marmoratus	Mayenne	ZMA.RenA_9080	1C 11T 1S_Cd	12
T. marmoratus	Mayenne	ZMA.RenA_9080	1C 11T 1S_Cd	12
T. marmoratus	Mayenne	ZMA.RenA_9081	1C 12T 1S_Cd	13
T. marmoratus	Mayenne	ZMA.RenA_9081	1C 11T 1S_Cd	12
T. marmoratus	Mayenne	ZMA.RenA_9081	1C 11T 1S_Cd	12
T. marmoratus	Mayenne	ZMA.RenA_9242	1C 11T 1S_Cd	12
T. marmoratus	Mayenne	ZMA.RenA_9242	1C 11T 1S_Cd	12
T. marmoratus	Mayenne	ZMA.RenA_9242	1C 11T 1S_Cd	12
T. marmoratus	Mayenne	ZMA.RenA_ 9266	1C 11T 1S_Cd	12
T. marmoratus	Mayenne	ZMA.RenA_9266	1C 12T 1S_Cd	13
T. marmoratus	Mayenne	ZMA.RenA_ 9266	1C 11T 1S_Cd	12
T. marmoratus	Rochechouart	ZMA.RenA_ 9151	1C 12T 1S_Cd	13
T. marmoratus	Rochechouart	ZMA.RenA_9151	1C 11T 0.5T/S 0.5S/Cd_Cd	12.5
T. marmoratus	Rochechouart	ZMA.RenA_9151	1C 11T 1S_Cd	12
T. marmoratus	Rochechouart	ZMA.RenA_ 9151	1C 11T 1S_Cd	12
T. ivanbureschi	Mersimbeleni	IBISS_G22464	1C 12T 1S_Cd	13
T. ivanbureschi	Mersimbeleni	IBISS_G22462	1C 12T 0.5T/S 0.5S/Cd_Cd	13.5
T. ivanbureschi	Mersimbeleni	IBISS_G22475	1C 13T 1S_Cd	14
T. ivanbureschi	Mersimbeleni	IBISS_G22470	1C 12T 1S_Cd	13
T. ivanbureschi	Mersimbeleni	IBISS_G22460	1C 12T 1S_Cd	13
T. ivanbureschi	Mersimbeleni	IBISS_G22467	1C 11T 0.5T/S 0.5S/Cd_Cd	12.5
T. ivanbureschi	Mersimbeleni	IBISS_G22468	1C 12T 1S_Cd	13
T. ivanbureschi	Mersimbeleni	IBISS_G22472	1C 12T 1S_Cd	13
T. ivanbureschi	Mersimbeleni	IBISS_G22459	1C 12T 1S_Cd	13
T. ivanbureschi	Mersimbeleni	IBISS_G22476	1C 12T 1S_Cd	13
T. ivanbureschi	Mersimbeleni	IBISS_G22466	1C 12T 1S_Cd	13
T. ivanbureschi	Mersimbeleni	IBISS_G22474	1C 12T 1S_Cd	13
T. ivanbureschi	Mersimbeleni	IBISS_G22473	1C 11T 1S_Cd	12
T. ivanbureschi	Mersimbeleni	IBISS_G22469	1C 12T 1S_Cd	13
T. ivanbureschi	Mersimbeleni	IBISS_G22471	1C 12T 1S_Cd	13

T. ivanbureschi	Mersimbeleni	IBISS_G22461	1C 12T 1S_Cd	13
T. ivanbureschi	Mersimbeleni	IBISS_G22477	1C 12T 1S_Cd	13
T. ivanbureschi	Mersimbeleni	IBISS_G22463	1C 12T 1S_Cd	13
T. ivanbureschi	Mersimbeleni	IBISS_G22465	1C 12T 1S_Cd	13
T. ivanbureschi	Reşadye	IBISS_G22502	1C 12T 1S_Cd	13
T. ivanbureschi	Reşadye	IBISS_G22507	1C 12T 1S_Cd	13
T. ivanbureschi	Reşadye	IBISS_G22504	1C 12T 1S_Cd	13
T. ivanbureschi	Reşadye	IBISS_G22506	1C 12T 1S_Cd	13
T. ivanbureschi	Reşadye	IBISS_G22516	1C 12T 1S_Cd	13
T. ivanbureschi	Reşadye	IBISS_G22512	1C 12T 1S_Cd	13
T. ivanbureschi	Reşadye	IBISS_G22501	1C 13T 1S_Cd	14
T. ivanbureschi	Reşadye	IBISS_G22505	1C 12T 1S_Cd	13
T. ivanbureschi	Reşadye	IBISS_G22503	1C 12T 1S_Cd	13
T. ivanbureschi	Reşadye	IBISS_G22508	1C 12T 1S_Cd	13
T. ivanbureschi	Reşadye	IBISS_G22514	1C 12T 1S_Cd	13
T. ivanbureschi	Reşadye	IBISS_G22509	1C 12T 1S_Cd	13
T. ivanbureschi	Reşadye	IBISS_G22500	1C 12T 1S_Cd	13
T. ivanbureschi	Reşadye	IBISS_G22510	1C 12T 1S_Cd	13
T. ivanbureschi	Reşadye	IBISS_G22519	1C 12T 1S_Cd	13
T. ivanbureschi	Reşadye	IBISS_G22518	1C 12T 1S_Cd	13
T. ivanbureschi	Reşadye	IBISS_G22517	1C 12T 1S_Cd	13
T. ivanbureschi	Reşadye	IBISS_G22515	1C 12T 1S_Cd	13
T. ivanbureschi	Reşadye	IBISS_G22513	1C 12T 1S_Cd	13
T. ivanbureschi	Reşadye	IBISS_G22511	1C 12T 1S_Cd	13
T. ivanbureschi	Serefiye	IBISS_G22439	1C 13T 1S_Cd	14
T. ivanbureschi	Serefiye	IBISS_G22457	1C 12T 1S_Cd	13
T. ivanbureschi	Serefiye	IBISS_G22454	1C 12T 1S_Cd	13
T. ivanbureschi	Serefiye	IBISS_G22442	1C 12T 1S_Cd	13
T. ivanbureschi	Serefiye	IBISS_G22440	1C 12T 0.5T/S 0.5S/Cd_Cd	13.5
T. ivanbureschi	Serefiye	IBISS_G22450	0.5C 12.5T 1S_Cd	13
T. ivanbureschi	Serefiye	IBISS_G22453	1C 12T 1S_Cd	13
T. ivanbureschi	Serefiye	IBISS_G22451	1C 12T 1S_Cd	13
T. ivanbureschi	Serefiye	IBISS_G22443	1C 12T 1S_Cd	13
T. ivanbureschi	Serefiye	IBISS_G22455	1C 12T 1S_Cd	13

T. ivanbureschi	Serefiye	IBISS_G22458	1C 12T 1S_Cd	13
T. ivanbureschi	Serefiye	IBISS_G22441	1C 12T 1S_Cd	13
T. ivanbureschi	Serefiye	IBISS_G22444	1C 12T 1S_Cd	13
T. ivanbureschi	Serefiye	IBISS_G22445	1C 13T 1S_Cd	14
T. ivanbureschi	Serefiye	IBISS_G22452	1C 12T 1S_Cd	13
T. ivanbureschi	Serefiye	IBISS_G22456	1C 12T 1S_Cd	13
T. ivanbureschi	Serefiye	IBISS_G22448	1C 12T 1S_Cd	13
T. ivanbureschi	Serefiye	IBISS_G22449	1C 12T 1S_Cd	13
T. ivanbureschi	Serefiye	IBISS_G22447	1C 12T 1S_Cd	13
T. ivanbureschi	Klaros	IBISS_G22527	1C 12T 1S_Cd	13
T. ivanbureschi	Klaros	IBISS_G22528	1C 11T 0.5T/S 0.5S/Cd_Cd	12.5
T. ivanbureschi	Klaros	IBISS_G22532	1C 11T 0.5T/S 0.5S/Cd_Cd	12.5
T. ivanbureschi	Klaros	IBISS_G22521	1C 12T 1S_Cd	13
T. ivanbureschi	Klaros	IBISS_G22526	1C 12T 1S_Cd	13
T. ivanbureschi	Klaros	IBISS_G22523	1C 12T 1S_Cd	13
T. ivanbureschi	Klaros	IBISS_G22522	1C 12T 1S_Cd	13
T. ivanbureschi	Klaros	IBISS_G22529	1C 12T 1S_Cd	13
T. ivanbureschi	Klaros	IBISS_G22525	1C 12T 1S_Cd	13
T. ivanbureschi	Klaros	IBISS_G22524	1C 12T 1S_Cd	13
T. ivanbureschi	Klaros	IBISS_G22520	1C 12T 1S_Cd	13
T. ivanbureschi	Klaros	IBISS_G22539	1C 12T 1S_Cd	13
T. ivanbureschi	Klaros	IBISS_G22534	1C 12T 1S_Cd	13
T. ivanbureschi	Klaros	IBISS_G22530	1C 12T 1S_Cd	13
T. ivanbureschi	Klaros	IBISS_G22538	1C 12T 1S_Cd	13
T. ivanbureschi	Klaros	IBISS_G22535	1C 12T 1S_Cd	13
T. ivanbureschi	Klaros	IBISS_G22533	1C 12T 1S_Cd	13
T. ivanbureschi	Klaros	IBISS_G22531	1C 12T 1S_Cd	13
T. ivanbureschi	Klaros	IBISS_G22536	1C 12T 1S_Cd	13
T. ivanbureschi	Kalecik	IBISS_G22492	1C 12T 1S_Cd	13
T. ivanbureschi	Kalecik	IBISS_G22484	1C 12T 1S_Cd	13
T. ivanbureschi	Kalecik	IBISS_G22488	1C 12T 1S_Cd	13
T. ivanbureschi	Kalecik	IBISS_G22490	1C 12T 1S_Cd	13
T. ivanbureschi	Kalecik	IBISS_G22491	1C 12T 1S_Cd	13
T. ivanbureschi	Kalecik	IBISS_G22489	1C 13T 1S_Cd	14

T. ivanbureschi	Kalecik	IBISS_G22493	1C 12T 1S_Cd	13
T. ivanbureschi	Kalecik	IBISS_G22480	1C 12T 1S_Cd	13
T. ivanbureschi	Kalecik	IBISS_G22487	1C 12T 1S_Cd	13
T. ivanbureschi	Kalecik	IBISS_G22479	1C 12T 0.5T/S 0.5S/Cd_Cd	13.5
T. ivanbureschi	Kalecik	IBISS_G22483	1C 12T 1S_Cd	13
T. ivanbureschi	Kalecik	IBISS_G22486	1C 12T 1S_Cd	13
T. ivanbureschi	Kalecik	IBISS_G22482	1C 12T 1S_Cd	13
T. ivanbureschi	Kalecik	IBISS_G22485	1C 13T 1S_Cd	14
T. ivanbureschi	Kalecik	IBISS_G22481	1C 12T 1S_Cd	13
T. ivanbureschi	Arifiye	IBISS_G22497	1C 12T 1S_Cd	13
T. ivanbureschi	Arifiye	IBISS_G22494	1C 13T 1S_Cd	14
T. ivanbureschi	Arifiye	IBISS_G22495	1C 12T 1S_Cd	13
T. ivanbureschi	Arifiye	IBISS_G22498	1C 13T 1S_Cd	14
T. ivanbureschi	Arifiye	IBISS_G22499	1C 12T 1S_Cd	13
T. ivanbureschi	Arifiye	IBISS_G22496	1C 12T 1S_Cd	13
T. ivanbureschi	Afyon	IBISS_G22882	1C 12T 1S_Cd	13
T. ivanbureschi	Afyon	IBISS_G22880	1C 12T 1S_Cd	13
T. ivanbureschi	Afyon	IBISS_G22877	1C 12T 1S_Cd	13
T. ivanbureschi	Afyon	IBISS_G22879	1C 13T 1S_Cd	14
T. ivanbureschi	Afyon	IBISS_G22883	1C 12T 0.5T/S 0.5S/Cd_Cd	13.5
T. ivanbureschi	Afyon	IBISS_G22881	1C 13T 1S_Cd	14
T. ivanbureschi	Afyon	IBISS_G22878	1C 12T 1S_Cd	13
T. ivanbureschi	Afyon	IBISS_G22876	1C 12T 1S_Cd	13
T. ivanbureschi	Afyon	IBISS_G22885	1C 12T 1S_Cd	13
T. ivanbureschi	Afyon	IBISS_G22886	1C 12T 1S_Cd	13
T. ivanbureschi	Afyon	IBISS_G22887	1C 12T 1S_Cd	13
T. ivanbureschi	Trabzon	IBISS_G22888	1C 12T 1S_Cd	13
T. ivanbureschi	Trabzon	IBISS_G22891	1C 12T 1S_Cd	13
T. ivanbureschi	Trabzon	IBISS_G22889	1C 12T 1S_Cd	13
T. ivanbureschi	Trabzon	IBISS_G22890	1C 12T 1S_Cd	13
T. ivanbureschi	Tosya	IBISS_G22853	1C 12T 1S_Cd	13
T. ivanbureschi	Tosya	IBISS_G22851	1C 13T 1S_Cd	14
T. ivanbureschi	Tosya	IBISS_G22839	1C 12T 1S_Cd	13
T. ivanbureschi	Tosya	IBISS_G22840	1C 12T 1S_Cd	13

T. ivanbureschi	Tosya	IBISS_G22842	1C 12T 1S_Cd	13
T. ivanbureschi	Tosya	IBISS_G22838	1C 12T 1S_Cd	13
T. ivanbureschi	Tosya	IBISS_G22844	1C 12T 1S_Cd	13
T. ivanbureschi	Tosya	IBISS_G22845	1C 12T 1S_Cd	13
T. ivanbureschi	Tosya	IBISS_G22846	1C 12T 1S_Cd	13
T. ivanbureschi	Tosya	IBISS_G22841	1C 12T 1S 0.5S/Cd_Cd	13
T. ivanbureschi	Tosya	IBISS_G22843	1C 12T 1S_Cd	13
T. ivanbureschi	Tosya	IBISS_G22855	1C 12T 1S_Cd	13
T. ivanbureschi	Tosya	IBISS_G22849	1C 12T 1S_Cd	13
T. ivanbureschi	Tosya	IBISS_G22847	1C 12T 1S_Cd	13
T. ivanbureschi	Tosya	IBISS_G22852	1C 12T 1S_Cd	13
T. ivanbureschi	Tosya	IBISS_G22854	1C 12T 1S_Cd	13
T. ivanbureschi	Tosya	IBISS_G22850	1C 12T 1S_Cd	13
T. ivanbureschi	Tosya	IBISS_G22848	1C 12T 1S_Cd	13
T. ivanbureschi	Bursa	IBISS_G22874	1C 12T 1S_Cd	13
T. ivanbureschi	Bursa	IBISS_G22869	1C 12T 1S_Cd	13
T. ivanbureschi	Bursa	IBISS_G22859	1C 12T 1S_Cd	13
T. ivanbureschi	Bursa	IBISS_G22865	1C 12T 1S_Cd	13
T. ivanbureschi	Bursa	IBISS_G22864	0.5C 12.5T 1S_Cd	13
T. ivanbureschi	Bursa	IBISS_G22856	1C 12T 1S_Cd	13
T. ivanbureschi	Bursa	IBISS_G22875	1C 12T 1S_Cd	13
T. ivanbureschi	Bursa	IBISS_G22858	1C 12T 1S_Cd	13
T. ivanbureschi	Bursa	IBISS_G22868	1C 12T 1S_Cd	13
T. ivanbureschi	Bursa	IBISS_G22863	1C 12T 1S_Cd	13
T. ivanbureschi	Bursa	IBISS_G22857	1C 12T 1S_Cd	13
T. ivanbureschi	Bursa	IBISS_G22860	1C 12T 1S_Cd	13
T. ivanbureschi	Bursa	IBISS_G22862	1C 12T 1S_Cd	13
T. ivanbureschi	Bursa	IBISS_G22861	1C 12T 1S_Cd	13
T. ivanbureschi	Bursa	IBISS_G22866	1C 12T 1S_Cd	13
T. ivanbureschi	Bursa	IBISS_G22867	1C 12T 1S_Cd	13
T. ivanbureschi	Bursa	IBISS_G22871	1C 12T 1S_Cd	13
T. ivanbureschi	Bursa	IBISS_G22870	1C 12T 1S_Cd	13
T. ivanbureschi	Bursa	IBISS_G22872	1C 12T 1S_Cd	13
T. ivanbureschi	Bursa	IBISS_G22873	1C 12T 1S_Cd	13

T. ivanbureschi	Guberevac	ZMA.RenA_ 8186	1C 12T 1S_Cd	13
T. ivanbureschi	Guberevac	ZMA.RenA_ 8186	1C 12T 1S_Cd	13
T. ivanbureschi	Guberevac	ZMA.RenA_ 8186	1C 12T 1S_Cd	13
T. ivanbureschi	Guberevac	ZMA.RenA_ 8186	1C 12T 1S 0.5S/Cd_Cd	13
T. ivanbureschi	Guberevac	ZMA.RenA_ 9116	1C 12T 1S_Cd	13
T. ivanbureschi	Guberevac	ZMA.RenA_ 9116	1C 12T 1S_Cd	13
T. ivanbureschi	Guberevac	ZMA.RenA_ 9116	1C 12T 1S_Cd	13
T. ivanbureschi	Guberevac	ZMA.RenA_ 9116	1C 12T 1S_Cd	13
T. ivanbureschi	Guberevac	ZMA.RenA_ 9116	1C 13T 1S_Cd	14
T. ivanbureschi	Istanbul	ZMA.RenA_9118	1C 13T 1S_Cd	14
T. ivanbureschi	Istanbul	ZMA.RenA_ 9118	1C 12T 1S_Cd	13
T. ivanbureschi	Istanbul	ZMA.RenA_ 9118	1C 12T 1S_Cd	13
T. ivanbureschi	Istanbul	ZMA.RenA_ 9118	1C 12T 1S_Cd	13
T. ivanbureschi	Istanbul	ZMA.RenA_ 9118	1C 13T 1S_Cd	14
T. ivanbureschi	Istanbul	ZMA.RenA_ 9118	1C 12T 1S_Cd	13
T. ivanbureschi	Istanbul	ZMA.RenA_ 9118	1C 12T 1S_Cd	13
T. ivanbureschi	Istanbul	ZMA.RenA_9118	1C 12T 1S_Cd	13
T. ivanbureschi	Istanbul	ZMA.RenA_9118	1C 13T 1S_Cd	14
T. ivanbureschi	Karlovo	ZMA.RenA_ 9125	1C 12T 1S_Cd	13
T. ivanbureschi	Karlovo	ZMA.RenA_9125	1C 12T 1S_Cd	13
T. ivanbureschi	Karlovo	ZMA.RenA_ 9125	1C 13T 1S_Cd	14
T. ivanbureschi	Trešnja	ZMA.RenA_9164	1C 12T 1S_Cd	13
T. ivanbureschi	Trešnja	ZMA.RenA_ 9164	1C 13T 1S_Cd	14
T. ivanbureschi	Trešnja	ZMA.RenA_ 9164	1C 12T 1S_Cd	13
T. ivanbureschi	Trešnja	ZMA.RenA_ 9164	1C 13T 1S_Cd	14
T. ivanbureschi	Trešnja	ZMA.RenA_9164	1C 12T 1S_Cd	13
T. ivanbureschi	Trešnja	ZMA.RenA_ 9164	1C 13T 1S_Cd	14
T. ivanbureschi	Trešnja	ZMA.RenA_ 9164	1C 12T 1S_Cd	13
T. ivanbureschi	Trešnja	ZMA.RenA_9164	1C 13T 1S_Cd	14
T. ivanbureschi	Trešnja	ZMA.RenA_ 9164	1C 13T 1S_Cd	14
T. ivanbureschi	Bartin	ZMA.RenA_7564	1C 12T 1S_Cd	13
T. ivanbureschi	Bartin	ZMA.RenA_7564	1C 12T 1S_Cd	13
T. ivanbureschi	Bartin	ZMA.RenA_7564	1C 13T 1S_Cd	14
T. ivanbureschi	Bartin	ZMA.RenA_7564	1C 12T 1S_Cd	13

T. ivanbureschi	Bartin	7ZMA.RenA_564	1C 12T 1S_Cd	13
T. ivanbureschi	Bartin	7ZMA.RenA_564	1C 12T 1S_Cd	13
T. ivanbureschi	Bartin	ZMA.RenA_7564	1C 12T 1S_Cd	13
T. ivanbureschi	Bartin	ZMA.RenA_7564	1C 12T 1S_Cd	13
T. ivanbureschi	Bartin	ZMA.RenA_7564	1C 12T 1S_Cd	13
T. ivanbureschi	Trešnja	IBISS_11c9	1C 13T 1S_Cd	14
T. ivanbureschi	Trešnja	IBISS_4c9	1C 13T 1S_Cd	14
T. ivanbureschi	Trešnja	IBISS_8c9	1C 12T 0.5T/S 0.5S/Cd_Cd	13.5
T. ivanbureschi	Trešnja	IBISS_7c9	1C 12T 1S_Cd	13
T. ivanbureschi	Trešnja	IBISS_3c9	1C 13T 1S_Cd	14
T. ivanbureschi	Trešnja	IBISS_5c9	1C 12T 1S_Cd	13
T. ivanbureschi	Trešnja	IBISS_13c9	1C 13T 1S_Cd	14
T. ivanbureschi	Trešnja	IBISS_6c9	1C 13T 1S_Cd	14
T. ivanbureschi	Trešnja	IBISS_9c9	1C 12T 1S_Cd	13
T. ivanbureschi	Trešnja	IBISS_1c9	1C 12T 1S_Cd	13
T. ivanbureschi	Trešnja	IBISS_16c9	1C 13T 1S_Cd	14
T. ivanbureschi	Trešnja	IBISS_35c9	1C 12T 1S_Cd	13
T. ivanbureschi	Trešnja	IBISS_54c9	1C 12T 1S_Cd	13
T. ivanbureschi	Trešnja	IBISS_33c9	1C 12T 1S_Cd	13
T. ivanbureschi	Trešnja	IBISS_51c9	1C 13T 1S_Cd	14
T. ivanbureschi	Trešnja	IBISS_5c9	1C 13T 1S_Cd	14
T. ivanbureschi	Trešnja	IBISS_48c9	1C 13T 1S_Cd	14
T. ivanbureschi	Trešnja	IBISS_42c9	1C 12T 1S 0.5S/Cd_Cd	13
T. ivanbureschi	Trešnja	IBISS_49c9	1C 12T 1S_Cd	13
T. ivanbureschi	Trešnja	IBISS_52c9	1C 13T 1S_Cd	14
T. ivanbureschi	Trešnja	IBISS_46c9	1C 12T 1S_Cd	13
T. ivanbureschi	Trešnja	IBISS_28c9	1C 12T 1S_Cd	13
T. ivanbureschi	Trešnja	IBISS_37c9	1C 13T 1S_Cd	14
T. ivanbureschi	Trešnja	IBISS_22c9	1C 13T 1S_Cd	14
T. ivanbureschi	Trešnja	IBISS_47c9	1C 12T 1S_Cd	13
T. ivanbureschi	Dafnohori	ZMA.RenA_9096	1C 13T 1S_Cd	14
T. ivanbureschi	Dafnohori	ZMA.RenA_9096	1C 13T 1S_Cd	14
T. ivanbureschi	Dafnohori	ZMA.RenA_9096	1C 12T 1S_Cd	13
T. ivanbureschi	Gornja Sabanta	ZMA.RenA_9113	1C 12T 1S_Cd	13

T. ivanbureschi	Karacaby	ZMA.RenA_9121	1C 12T 1S_Cd	13
T. ivanbureschi	Karacaby	ZMA.RenA_9121	1C 13T 1S_Cd	14
T. ivanbureschi	Karacaby	ZMA.RenA_9121	1C 12T 1S_Cd	13
T. ivanbureschi	Mitrašinci	ZMA.RenA_9140	1C 12T 1S_Cd	14
T. ivanbureschi	Mitrašinci	ZMA.RenA_9140	1C 12T 1S_Cd	13
T. ivanbureschi	Mitrašinci	ZMA.RenA_9140	1C 11T 1S_Cd	12
T. ivanbureschi	Mitrašinci	ZMA.RenA_9140	1C 13T 1S_Cd	13
T. ivanbureschi	Mitrašinci	ZMA.RenA_9140	1C 12T 1S_Cd	13
T. ivanbureschi	Mitrašinci	ZMA.RenA_9140	1C 12T 0.5T/S 0.5S/Cd_Cd	13
T. ivanbureschi	Mitrašinci	ZMA.RenA_9140	1C 12T 1S_Cd	13
T. ivanbureschi	Mitrašinci	ZMA.RenA_9140	1C 12T 1S_Cd	13
T. ivanbureschi	Mitrašinci	ZMA.RenA_9140	1C 12T 1S_Cd	13
T. ivanbureschi	Mitrašinci	ZMA.RenA_9140	1C 13T 1S_Cd	14
T. ivanbureschi	Mitrašinci	ZMA.RenA_9140	1C 13T 1S_Cd	14
T. ivanbureschi	Mitrašinci	ZMA.RenA_9140	0.5C/T 12.5T 1S_Cd	13
T. ivanbureschi	Resavica Pećina	ZMA.RenA_9150	1C 12T 0.5T/S 0.5S/Cd_Cd	13.5
T. ivanbureschi	Resavica Pećina	ZMA.RenA_9150	1C 12T 1S_Cd	13
T. ivanbureschi	Resavica Pećina	ZMA.RenA_9150	1C 13T 1S_Cd	14
T. ivanbureschi	Sevlievo	ZMA.RenA_9154	1C 12T 1S_Cd	13
T. ivanbureschi	Sevlievo	ZMA.RenA_9154	1C 12T 1S_Cd	13
T. ivanbureschi	Sićevac	ZMA.RenA_9157	1C 13T 1S_Cd	14
T. ivanbureschi	Sićevac	ZMA.RenA_9157	1C 12T 1S_Cd	13
T. ivanbureschi	Sićevac	ZMA.RenA_9157	1C 12T 1S_Cd	13
T. ivanbureschi	Sićevac	ZMA.RenA_9157	1C 12T 1S_Cd	13
T. ivanbureschi	Vitanovac	ZMA.RenA_46933	1C 12T 1S_Cd	13
T. ivanbureschi	Vitanovac	ZMA.RenA_46933	1C 12T 1S_Cd	13
T. ivanbureschi	Vitanovac	ZMA.RenA_46933	1C 13T 1S_Cd	14
T. ivanbureschi	Vitanovac	ZMA.RenA_46933	1C 12T 1S_Cd	13
T. ivanbureschi	Đurinci	ZMA.RenA_9099	1C 12T 1S_Cd	14
T. ivanbureschi	Đurinci	ZMA.RenA_9099	1C 13T 1S_Cd	14
T. ivanbureschi	Kentriko	ZMA.RenA_9127	1C 13T 1S_Cd	14
T. ivanbureschi	Kentriko	ZMA.RenA_9127	1C 12T 1S_Cd	13
T. ivanbureschi	Kentriko	ZMA.RenA_9127	1C 12T 1S_Cd	13
T. ivanbureschi	Kentriko	ZMA.RenA_9127	1C 12T 1S_Cd	13

T. ivanbureschi	Kentriko	ZMA.RenA_9127	1C 12T 1S_Cd	13
T. ivanbureschi	Kentriko	ZMA.RenA_9127	1C 12T 1S_Cd	13
T. ivanbureschi	Kentriko	ZMA.RenA_9127	1C 12T 1S_Cd	13
T. ivanbureschi	Kentriko	ZMA.RenA_9127	1C 12T 1S_Cd	13
T. ivanbureschi	Kentriko	ZMA.RenA_9127	1C 13T 1S_Cd	14
T. ivanbureschi	Kentriko	ZMA.RenA_9127	1C 13T 1S_Cd	14
T. ivanbureschi	Kentriko	ZMA.RenA_9127	1C 13T 1S_Cd	14
T. ivanbureschi	Kentriko	ZMA.RenA_9127	1C 12T 1S_Cd	13
T. ivanbureschi	Kentriko	ZMA.RenA_9127	1C 13T 1S_Cd	14
T. ivanbureschi	Levski	ZMA.RenA_9134	1C 12T 1S_Cd	13
T. ivanbureschi	Levski	ZMA.RenA_9134	1C 12T 1S_Cd	13
T. ivanbureschi	Levski	ZMA.RenA_9134	1C 12T 0.5T/S 0.5S/Cd_Cd	13.5
T. ivanbureschi	Levski	ZMA.RenA_9134	1C 12T 1S_Cd	13
T. ivanbureschi	Levski	ZMA.RenA_9134	1C 12T 1S_Cd	13
T. ivanbureschi	Levski	ZMA.RenA_9134	1C 13T 1S_Cd	14
T. ivanbureschi	Levski	ZMA.RenA_9134	1C 12T 1S_Cd	13
T. ivanbureschi	Levski	ZMA.RenA_9134	1C 13T 1S_Cd	13
T. ivanbureschi	Levski	ZMA.RenA_9134	1C 12T 1S_Cd	13
T. ivanbureschi	Rakovski	ZMA.RenA_9149	1C 12T 1S_Cd	13
T. ivanbureschi	Rakovski	ZMA.RenA_9149	1C 12T 1S_Cd	13
T. ivanbureschi	Rakovski	ZMA.RenA_9149	1C 13T 1S_Cd	14
T. ivanbureschi	Rakovski	ZMA.RenA_9149	1C 12T 0.5T/S 0.5S/Cd_Cd	13.5
T. ivanbureschi	Vitanovac	ZMA.RenA_9169	1C 12T 1S_Cd	13
T. ivanbureschi	Vitanovac	ZMA.RenA_9169	1C 13T 0.5T/S 0.5S/Cd_Cd	14.5
T. ivanbureschi	Vitanovac	ZMA.RenA_9169	0C 14T 1S_Cd	14
T. ivanbureschi	Vitanovac	ZMA.RenA_9169	1C 12T 0.5T/S 0.5S/Cd_Cd	13.5
T. ivanbureschi	Vitanovac	ZMA.RenA_9169	1C 12T 1S_Cd	13
T. ivanbureschi	Aranđelovac	ZMA.RenA_9086	1C 12T 1S_Cd	13
T. ivanbureschi	Aranđelovac	ZMA.RenA_9086	1C 12T 1S_Cd	13
T. ivanbureschi	Aranđelovac	ZMA.RenA_9086	1C 12T 1S_Cd	13
T. ivanbureschi	Aranđelovac	ZMA.RenA_9086	1C 12T 1S_Cd	13
T. ivanbureschi	Aranđelovac	ZMA.RenA_9086	1C 12T 1S_Cd	13
T. ivanbureschi	Aranđelovac	ZMA.RenA_9086	1C 12T 0.5T/S 0.5S/Cd_Cd	13
T. ivanbureschi	Aranđelovac	ZMA.RenA_9086	1C 12T 1S_Cd	13

T. ivanbureschi	Aranđelovac	ZMA.RenA_9086	1C 12T 1S_Cd	13
T. ivanbureschi	Adapazari	ZMA.RenA_9082	1C 12T 1S_Cd	13
T. ivanbureschi	Adapazari	ZMA.RenA_9082	1C 12T 1S_Cd	13
T. ivanbureschi	Adapazari	ZMA.RenA_9082	1C 12T 1S_Cd	13
T. ivanbureschi	Adapazari	ZMA.RenA_9082	1C 12T 1S_Cd	13
T. ivanbureschi	Adapazari	ZMA.RenA_9082	1C 12T 1S_Cd	13
T. ivanbureschi	Adapazari	ZMA.RenA_9082	1C 12T 1S_Cd	13
T. ivanbureschi	Bigla	ZMA.RenA_9092	1C 12T 1S_Cd	13
T. ivanbureschi	Bigla	ZMA.RenA_9092	1C 12T 1S_Cd	13
T. ivanbureschi	Bigla	ZMA.RenA_9092	1C 12T 1S_Cd	13
T. ivanbureschi	Bigla	ZMA.RenA_9092	1C 12T 1S_Cd	13
T. ivanbureschi	Bigla	ZMA.RenA_9092	1C 12T 1S_Cd	13
T. ivanbureschi	Grivac	ZMA.RenA_9115	1C 12T 1S_Cd	13
T. ivanbureschi	Grivac	ZMA.RenA_9115	1C 12T 1S_Cd	13
T. ivanbureschi	Grivac	ZMA.RenA_9115	1C 12T 1S_Cd	13
T. ivanbureschi	Grivac	ZMA.RenA_9115	1C 12T 1S_Cd	13
T. ivanbureschi	Grivac	ZMA.RenA_9115	1C 11T 1S_Cd	12
T. ivanbureschi	Grivac	ZMA.RenA_9115	1C 12T 1S_Cd	13
T. ivanbureschi	Grivac	ZMA.RenA_9115	1C 12T 1S_Cd	13
T. ivanbureschi	Grivac	ZMA.RenA_9115	1C 12T 1S_Cd	13
T. ivanbureschi	Grivac	ZMA.RenA_9115	1C 12T 1S_Cd	13
T. karelinii	Georgia	IBISS_G22246	1C 12T 1S_Cd	13
T. karelinii	Georgia	IBISS_G22247	1C 12T 1S_Cd	13
T. karelinii	Georgia	IBISS_G22245	1C 12T 1S_Cd	13
T. karelinii	Georgia	IBISS_G22242	1C 12T 1S_Cd	13
T. karelinii	Georgia	IBISS_G22243	1C 12T 1S_Cd	13
T. karelinii	Georgia	IBISS_G22244	1C 12T 1S_Cd	13
T. karelinii	Kutuzovsko lake	IBISS_G22829	1C 12T 1S_Cd	13
T. karelinii	Kutuzovsko lake	IBISS_G22832	1C 12T 1S_Cd	13
T. karelinii	Kutuzovsko lake	IBISS_G22827	1C 12T 1S_Cd	13
T. karelinii	Kutuzovsko lake	IBISS_G22822	1C 12T 1S_Cd	13
T. karelinii	Kutuzovsko lake	IBISS_G22823	1C 12T 1S_Cd	13
T. karelinii	Kutuzovsko lake	IBISS_G22837	1C 12T 1S_Cd	13
T. karelinii	Kutuzovsko lake	IBISS_G22824	1C 12T 1S_Cd	13

T. karelinii	Kutuzovsko lake	IBISS_G22828	1C 12T 1S_Cd	13
T. karelinii	Kutuzovsko lake	IBISS_G22831	1C 12T 1S_Cd	13
T. karelinii	Kutuzovsko lake	IBISS_G22830	1C 12T 1S_Cd	13
T. karelinii	Kutuzovsko lake	IBISS_G22826	1C 12T 1S_Cd	13
T. karelinii	Kutuzovsko lake	IBISS_G22825	1C 12T 1S_Cd	13
T. karelinii	Kutuzovsko lake	IBISS_G22833	1C 12T 1S_Cd	13
T. karelinii	Kutuzovsko lake	IBISS_G22835	1C 12T 1S_Cd	13
T. karelinii	Kutuzovsko lake	IBISS_G22836	1C 12T 1S_Cd	13
T. karelinii	Tabasaranskii	IBISS_G22809	1C 12T 1S_Cd	13
T. karelinii	Tabasaranskii	IBISS_G22802	1C 12T 1S_Cd	13
T. karelinii	Tabasaranskii	IBISS_G22805	1C 11T 0.5T/S 0.5S/Cd_Cd	12.5
T. karelinii	Tabasaranskii	IBISS_G22813	1C 12T 1S_Cd	13
T. karelinii	Tabasaranskii	IBISS_G22811	1C 12T 1S_Cd	13
T. karelinii	Tabasaranskii	IBISS_G22807	1C 12T 1S_Cd	13
T. karelinii	Tabasaranskii	IBISS_G22806	1C 12T 1S_Cd	13
T. karelinii	Tabasaranskii	IBISS_G22808	1C 12T 1S_Cd	13
T. karelinii	Tabasaranskii	IBISS_G22804	1C 12T 1S_Cd	13
T. karelinii	Tabasaranskii	IBISS_G22821	1C 12T 1S_Cd	13
T. karelinii	Tabasaranskii	IBISS_G22818	1C 12T 1S_Cd	13
T. karelinii	Tabasaranskii	IBISS_G22820	1C 12T 1S_Cd	13
T. karelinii	Tabasaranskii	IBISS_G22817	1C 12T 1S_Cd	13
T. karelinii	Tabasaranskii	IBISS_G22816	1C 12T 1S_Cd	13
T. karelinii	Tabasaranskii	IBISS_G22814	1C 12T 1S_Cd	13
T. karelinii	Tabasaranskii	IBISS_G22819	1C 12T 1S_Cd	13
T. karelinii	Tabasaranskii	IBISS_G22810	1C 12T 1S_Cd	13
T. karelinii	Tabasaranskii	IBISS_G22815	1C 12T 1S_Cd	13
T. karelinii	Dizabad	ZMA.RenA_2389	1C 12T 1S_Cd	13
T. karelinii	Dizabad	ZMA.RenA_2390	1C 13T 1S_Cd	14
T. karelinii	Akhaldaba	ZMA.RenA_9343	1C 13T 1S_Cd	14
T. karelinii	Akhaldaba	ZMA.RenA_ 9343	1C 12T 1S_Cd	13
T. macedonicus	Rataje	IBISS_57c1	0.5C/T 13.5T 1S_Cd	14
T. macedonicus	Rataje	IBISS_47c1	1C 13T 1S_Cd	14
T. macedonicus	Rataje	IBISS_30c1	1C 13T 1S_Cd	14
T. macedonicus	Rataje	IBISS_43c1	1C 13T 1S_Cd	14

T. macedonicus	Rataje	IBISS_49c1	1C 13T 1S_Cd	14
T. macedonicus	Rataje	IBISS_28c1	1C 13T 1S_Cd	14
T. macedonicus	Rataje	IBISS_37c1	1C 13T 1S_Cd	14
T. macedonicus	Rataje	IBISS_54c1	1C 13T 1S_Cd	14
T. macedonicus	Rataje	IBISS_36c1	1C 13T 1S_Cd	14
T. macedonicus	Rataje	IBISS_41c1	1C 13T 1S_Cd	14
T. macedonicus	Rataje	IBISS_38c1	1C 13T 0.5T/S 0.5S/Cd_Cd	14.5
T. macedonicus	Rataje	IBISS_53c1	1C 13T 1S_Cd	14
T. macedonicus	Rataje	IBISS_51c1	1C 13T 1S_Cd	14
T. macedonicus	Rataje	IBISS_33c1	1C 13T 1S_Cd	14
T. macedonicus	Rataje	IBISS_55c1	1C 13T 1S_Cd	14
T. macedonicus	Rataje	IBISS_34c1	1C 13T 1S_Cd	14
T. macedonicus	Rataje	IBISS_32c1	1C 13T 1S 0.5S/Cd_Cd	13
T. macedonicus	Rataje	IBISS_42c1	1C 13T 1S_Cd	14
T. macedonicus	Rataje	IBISS_44c1	1C 13T 1S_Cd	14
T. macedonicus	Rataje	IBISS_45c1	1C 12T 1S_Cd	13
T. macedonicus	Rataje	IBISS_52c1	1C 13T 1S_Cd	14
T. macedonicus	Rataje	IBISS_48c1	1C 13T 1S_Cd	14
T. macedonicus	Rataje	IBISS_31c1	1C 13T 1S_Cd	14
T. macedonicus	Rataje	IBISS_39c1	1C 13T 1S_Cd	14
T. macedonicus	Rataje	IBISS_35c1	1C 13T 1S_Cd	14
T. macedonicus	Rataje	IBISS_46c1	1C 13T 1S_Cd	14
T. macedonicus	Rataje	IBISS_29c1	1C 13T 1S_Cd	14
T. macedonicus	Rataje	IBISS_50c1	1C 13T 1S_Cd	13
T. macedonicus	Rataje	IBISS_56c1	1C 13T 1S_Cd	14
T. macedonicus	Rataje	IBISS_13c1	1C 13T 1S_Cd	14
T. macedonicus	Rataje	IBISS_11c1	1C 13T 1S_Cd	14
T. macedonicus	Rataje	IBISS_9c1	1C 12T 0.5T/S 0.5S/Cd_Cd	13.5
T. macedonicus	Rataje	IBISS_4c1	1C 13T 1S_Cd	14
T. macedonicus	Rataje	IBISS_26c1	1C 13T 1S_Cd	14
T. macedonicus	Rataje	IBISS_27c1	1C 13T 1S_Cd	14
T. macedonicus	Rataje	IBISS_1c1	1C 13T 1S_Cd	14
T. macedonicus	Rataje	IBISS_3c1	1C 13T 1S_Cd	14
T. macedonicus	Rataje	IBISS_20c1	1C 13T 1S_Cd	14

T. macedonicus	Rataje	IBISS_18c1	1C 13T 1S_Cd	14
T. macedonicus	Rataje	IBISS_10c1	1C 13T 1S_Cd	14
T. macedonicus	Rataje	IBISS_16c1	1C 13T 1S_Cd	14
T. macedonicus	Rataje	IBISS_7c1	1C 13T 1S_Cd	14
T. macedonicus	Rataje	IBISS_5c1	1C 13T 1S_Cd	14
T. macedonicus	Rataje	IBISS_25c1	1C 13T 1S_Cd	14
T. macedonicus	Rataje	IBISS_19c1	1C 13T 1S_Cd	14
T. macedonicus	Rataje	IBISS_2c1	1C 12T 1S_Cd	13
T. macedonicus	Rataje	IBISS_8c1	1C 13T 1S_Cd	14
T. macedonicus	Rataje	IBISS_14c1	1C 13T 1S_Cd	14
T. macedonicus	Rataje	IBISS_17c1	1C 13T 1S_Cd	14
T. macedonicus	Rataje	IBISS_24c1	1C 13T 1S_Cd	14
T. macedonicus	Rataje	IBISS_15c1	1C 13T 1S_Cd	14
T. macedonicus	Rataje	IBISS_23c1	1C 13T 1S_Cd	14
T. macedonicus	Rataje	IBISS_21c1	1C 13T 1S_Cd	14
T. macedonicus	Rataje	IBISS_6c1	1C 12T 1S_Cd	13
T. macedonicus	Rataje	IBISS_22c1	1C 13T 1S_Cd	14
T. macedonicus	Galičica	IBISS_10c5	1C 13T 1S_Cd	14
T. macedonicus	Galičica	IBISS_1c5	1C 13T 1S_Cd	14
T. macedonicus	Galičica	IBISS_22c5	1C 13T 1S_Cd	14
T. macedonicus	Galičica	IBISS_23c5	1C 13T 1S_Cd	14
T. macedonicus	Galičica	IBISS_2c5	1C 13T 1S_Cd	14
T. macedonicus	Galičica	IBISS_28c5	1C 13T 1S_Cd	14
T. macedonicus	Galičica	IBISS_13c5	1C 13T 1S_Cd	14
T. macedonicus	Galičica	IBISS_30c5	1C 13T 1S_Cd	14
T. macedonicus	Galičica	IBISS_26c5	1C 13T 1S_Cd	14
T. macedonicus	Galičica	IBISS_15c5	1C 13T 1S_Cd	14
T. macedonicus	Galičica	IBISS_19c5	1C 13T 1S_Cd	14
T. macedonicus	Galičica	IBISS_8c5	1C 13T 1S_Cd	14
T. macedonicus	Galičica	IBISS_16c5	1C 13T 0.5T/S 0.5S/Cd_Cd	14.5
T. macedonicus	Galičica	IBISS_25c5	1C 13T 1S_Cd	14
T. macedonicus	Galičica	IBISS_21c5	1C 13T 1S_Cd	14
T. macedonicus	Todorovce	IBISS_џ18931	1C 13T 1S_Cd	14
T. macedonicus	Todorovce	IBISS_џ18948	1C 13T 1S_Cd	14

T. macedonicus	Todorovce	IBISS_џ18945	1C 13T 1S_Cd	13.5
T. macedonicus	Todorovce	IBISS_џ18944	1C 13T 1S_Cd	14
T. macedonicus	Todorovce	IBISS_џ18933	1C 13T 1S_Cd	14
T. macedonicus	Todorovce	IBISS_џ18939	1C 13T 1S_Cd	14
T. macedonicus	Todorovce	IBISS_џ18941	1C 13T 1S_Cd	14
T. macedonicus	Todorovce	IBISS_џ18942	1C 13T 1S_Cd	14
T. macedonicus	Todorovce	IBISS_џ18934	1C 13T 1S_Cd	14
T. macedonicus	Todorovce	IBISS_џ18943	1C 13T 1S_Cd	14
T. macedonicus	Todorovce	IBISS_џ18929	1C 13T 1S_Cd	14
T. macedonicus	Todorovce	IBISS_џ18923	1C 13T 1S_Cd	14
T. macedonicus	Todorovce	IBISS_џ18924	1C 13T 1S_Cd	14
T. macedonicus	Todorovce	IBISS_џ18938	1C 13T 1S_Cd	14
T. macedonicus	Todorovce	IBISS_џ18940	1C 13T 1S_Cd	14
T. macedonicus	Divčibare	IBISS_G23012	1C 13T 1S_Cd	14
T. macedonicus	Divčibare	IBISS_G23007	1C 13T 1S_Cd	14
T. macedonicus	Divčibare	IBISS_G23008	1C 13T 1S_Cd	14
T. macedonicus	Divčibare	IBISS_G23009	1C 13T 1S_Cd	14
T. macedonicus	Divčibare	IBISS_G23003	1C 13T 1S_Cd	14
T. macedonicus	Divčibare	IBISS_G23002	1C 12T 1S_Cd	13
T. macedonicus	Divčibare	IBISS_G23004	1C 13T 1S_Cd	14
T. macedonicus	Divčibare	IBISS_G23011	1C 13T 1S_Cd	14
T. macedonicus	Divčibare	IBISS_G23010	1C 13T 1S_Cd	14
T. macedonicus	Divčibare	IBISS_G23006	1C 12T 1S_Cd	13
T. macedonicus	Divčibare	IBISS_G23001	1C 13T 1S_Cd	14
T. macedonicus	Divčibare	IBISS_G23000	1C 13T 1S_Cd	14
T. macedonicus	Divčibare	IBISS_G22999	1C 13T 1S_Cd	14
T. macedonicus	Divčibare	IBISS_G22998	1C 13T 1S_Cd	14
T. macedonicus	Divčibare	IBISS_G22995	1C 13T 1S_Cd	14
T. macedonicus	Divčibare	IBISS_G22993	1C 13T 1S_Cd	14
T. macedonicus	Divčibare	IBISS_G22997	1C 13T 1S_Cd	14
T. macedonicus	Divčibare	IBISS_G22996	1C 12T 1S_Cd	13
T. macedonicus	Divčibare	IBISS_G22994	1C 13T 1S_Cd	14
T. macedonicus	Divčibare	IBISS_G23005	1C 12T 1S_Cd	13
T. macedonicus	Višegrad	ZMA.RenA_9244	1C 13T 1S_Cd	14

T. macedonicus	Višegrad	ZMA.RenA_9244	1C 13T 1S_Cd	14
T. macedonicus	Stanišinci	ZMA.RenA_9158	1C 13T 1S_Cd	14
T. macedonicus	Stanišinci	ZMA.RenA_9158	1C 13T 1S_Cd	14
T. macedonicus	Stanišinci	ZMA.RenA_9158	1C 13T 1S_Cd	14
T. macedonicus	Stanišinci	ZMA.RenA_9158	1C 13T 1S_Cd	14
T. macedonicus	Ano Kalliniki	ZMA.RenA_ 9085	1C 13T 1S_Cd	14
T. macedonicus	Ano Kalliniki	ZMA.RenA_ 9085	1C 13T 1S_Cd	14
T. macedonicus	Ano Kalliniki	ZMA.RenA_9085	1C 13T 0.5T/S 0.5S/Cd_Cd	13.5
T. macedonicus	Ano Kalliniki	ZMA.RenA_ 9085	1C 13T 1S_Cd	14
T. macedonicus	Ano Kalliniki	ZMA.RenA_ 9085	1C 13T 1S_Cd	14
T. macedonicus	Ano Kalliniki	ZMA.RenA_ 9085	1C 13T 1S_Cd	14
T. macedonicus	Ano Kalliniki	ZMA.RenA_9085	1C 13T 1S_Cd	14
T. macedonicus	Karan	ZMA.RenA_ 9122	1C 13T 1S_Cd	14
T. macedonicus	Karan	ZMA.RenA_ 9122	1C 13T 1S_Cd	14
T. macedonicus	Karan	ZMA.RenA_ 9122	1C 13T 1S_Cd	14
T. macedonicus	Manastir Tavna	ZMA.RenA_9162	1C 14T 1S_Cd	15
T. macedonicus	Manastir Tavna	ZMA.RenA_9162	1C 13T 1S_Cd	14
T. macedonicus	Manastir Tavna	ZMA.RenA_ 9162	1C 12T 1S_Cd	13
T. macedonicus	Manastir Tavna	ZMA.RenA_9162	1C 13T 1S_Cd	14
T. macedonicus	Manastir Tavna	ZMA.RenA_9162	1C 13T 1S_Cd	14
T. macedonicus	Manastir Tavna	ZMA.RenA_ 9162	1C 13T 1S_Cd	14
T. macedonicus	Manastir Tavna	ZMA.RenA_ 9162	1C 13T 1S_Cd	14
T. macedonicus	Višegrad	ZMA.RenA_ 9168	1C 13T 1S_Cd	14
T. macedonicus	Višegrad	ZMA.RenA_ 9168	1C 13T 1S_Cd	14
T. macedonicus	Višegrad	ZMA.RenA_9168	1C 13T 0.5T/S 0.5S/Cd_Cd	13.5
T. macedonicus	Višegrad	ZMA.RenA_ 9168	1C 13T 1S_Cd	14
T. macedonicus	Višegrad	ZMA.RenA_9168	1C 12T 1S_Cd	13
T. macedonicus	Višegrad	ZMA.RenA_ 9168	1C 13T 1S_Cd	14
T. macedonicus	Višegrad	ZMA.RenA_ 9168	1C 13T 1S_Cd	14
T. macedonicus	Višegrad	ZMA.RenA_ 9168	1C 13T 1S_Cd	14
T. macedonicus	Višegrad	ZMA.RenA_ 9168	1C 13T 1S_Cd	14
T. macedonicus	Višegrad	ZMA.RenA_ 9168	1C 12T 1S_Cd	13
T. macedonicus	Višegrad	ZMA.RenA_9168	1C 13T 0.5T/S 0.5S/Cd_Cd	13.5
T. macedonicus	Višegrad	ZMA.RenA_ 9168	1C 14T 1S_Cd	15

T. macedonicus	Gornja Čađavica	ZMA.RenA_9112	1C 14T 1S_Cd	15
T. macedonicus	Gornja Čadjavica	ZMA.RenA_9112	1C 14T 0.5T/S 0.5S/Cd_Cd	14.5
T. macedonicus	Gornja Čadjavica	ZMA.RenA_9112	1C 14T 1S_Cd	15
T. macedonicus	Gornja Čadjavica	ZMA.RenA_9112	1C 13T 1S_Cd	14
T. macedonicus	Gornja Čadjavica	ZMA.RenA_9112	1C 15T 1S_Cd	16
T. macedonicus	Gornja Čadjavica	ZMA.RenA_9112	1C 13T 1S_Cd	14
T. macedonicus	Gornja Čadjavica	ZMA.RenA_9112	1C 13T 1S_Cd	14
T. macedonicus	Gornja Čadjavica	ZMA.RenA_9112	1C 13T 1S_Cd	14
T. macedonicus	Gornja Čadjavica	ZMA.RenA_9112	1C 15T 1S_Cd	16
T. macedonicus	Karan	ZMA.RenA_9123	1C 13T 1S_Cd	14
T. macedonicus	Karan	ZMA.RenA_9123	1C 13T 1S_Cd	14
T. macedonicus	Karan	ZMA.RenA_9124	1C 13T 1S_Cd	14
T. macedonicus	Lučane	ZMA.RenA_9137	1C 13T 1S_Cd	14
T. macedonicus	Lučane	ZMA.RenA_9137	1C 13T 1S_Cd	14
T. macedonicus	Lučane	ZMA.RenA_9137	1C 12T 1S_Cd	13
T. macedonicus	Lučane	ZMA.RenA_9137	1C 13T 1S_Cd	14
T. macedonicus	Lučane	ZMA.RenA_9137	1C 12T 1S_Cd	13
T. macedonicus	Lučane	ZMA.RenA_9137	1C 13T 1S_Cd	14
T. macedonicus	Lučane	ZMA.RenA_9137	1C 13T 1S_Cd	14
T. macedonicus	Lučane	ZMA.RenA_9137	1C 12T 0.5T/S 0.5S/Cd_Cd	13.5
T. macedonicus	Lučane	ZMA.RenA_9137	1C 12T 1S_Cd	13
T. macedonicus	Lučane	ZMA.RenA_9137	1C 13T 1S_Cd	14
T. macedonicus	Lučane	ZMA.RenA_9137	1C 13T 1S_Cd	14
T. macedonicus	Lučane	ZMA.RenA_9137	1C 13T 1S_Cd	14
T. macedonicus	Probistip	ZMA.RenA_9147	1C 12T 1S_Cd	13
T. macedonicus	Probistip	ZMA.RenA_9147	1C 12T 1S_Cd	13
T. macedonicus	Probistip	ZMA.RenA_9147	1C 12T 1S_Cd	13
T. macedonicus	Probistip	ZMA.RenA_9147	1C 12T 1S_Cd	13
T. macedonicus	Divčibare	ZMA.RenA_9098	1C 13T 1S_Cd	14
T. macedonicus	Divčibare	ZMA.RenA_9098	1C 13T 1S_Cd	14
T. macedonicus	Divčibare	ZMA.RenA_9098	1C 12T 1S_Cd	13
T. macedonicus	Divčibare	ZMA.RenA_9098	1C 13T 1S_Cd	14
T. macedonicus	Divčibare	ZMA.RenA_9098	1C 13T 1S_Cd	14
T. macedonicus	Divčibare	ZMA.RenA_9098	1C 13T 1S_Cd	14

T. macedonicus	Divčibare	ZMA.RenA_9098	1C 13T 1S_Cd	14
T. macedonicus	Divčibare	ZMA.RenA_9098	1C 13T 1S_Cd	14
T. macedonicus	Divčibare	ZMA.RenA_9098	1C 13T 1S_Cd	14
T. macedonicus	Divčibare	ZMA.RenA_9098	1C 13T 1S_Cd	14
T. macedonicus	Divčibare	ZMA.RenA_9098	1C 13T 1S_Cd	14
T. macedonicus	Grčak	ZMA.RenA_ 9114	1C 13T 1S_Cd	14
T. macedonicus	Grčak	ZMA.RenA_ 9114	1C 13T 1S_Cd	14
T. macedonicus	Grčak	ZMA.RenA_ 9114	1C 12T 1S_Cd	13
T. macedonicus	Grčak	ZMA.RenA_9114	1C 12T 1S_Cd	13
T. macedonicus	Grčak	ZMA.RenA_9114	1C 12T 1S_Cd	13
T. macedonicus	Grčak	ZMA.RenA_ 9114	1C 13T 1S_Cd	14
T. macedonicus	Grčak	ZMA.RenA_ 9114	1C 13T 1S_Cd	14
T. macedonicus	Grčak	ZMA.RenA_9114	1C 12T 1S_Cd	13
T. carnifex	Podstrmec	IBISS_25c11	1C 13T 1S_Cd	14
T. carnifex	Podstrmec	IBISS_13c11	1C 13T 1S_Cd	14
T. carnifex	Podstrmec	IBISS_24c11	1C 13T 1S_Cd	14
T. carnifex	Podstrmec	IBISS_22c11	1C 13T 1S_Cd	14
T. carnifex	Podstrmec	IBISS_14c11	1C 13T 1S_Cd	14
T. carnifex	Podstrmec	IBISS_17c11	1C 13T 1S_Cd	14
T. carnifex	Podstrmec	IBISS_19c11	1C 13T 1S_Cd	14
T. carnifex	Podstrmec	IBISS_18c11	1C 13T 1S_Cd	14
T. carnifex	Podstrmec	IBISS_15c11	1C 12T 0.5T/S 0.5S/Cd_Cd	13.5
T. carnifex	Podstrmec	IBISS_23c11	1C 13T 1S_Cd	14
T. carnifex	Podstrmec	IBISS_14c11	1C 13T 1S_Cd	14
T. carnifex	Podstrmec	IBISS_11c11	1C 12T 1S_Cd	13
T. carnifex	Podstrmec	IBISS_13c11	1C 13T 1S_Cd	14
T. carnifex	Podstrmec	IBISS_12c11	1C 13T 1S_Cd	14
T. carnifex	Podstrmec	IBISS_18c11	1C 13T 1S_Cd	14
T. carnifex	Podstrmec	IBISS_17c11	1C 13T 1S_Cd	14
T. carnifex	Podstrmec	IBISS_19c11	1C 13T 1S_Cd	14
T. carnifex	Podstrmec	IBISS_15c11	1C 13T 1S_Cd	14
T. carnifex	Podstrmec	IBISS_16c11	1C 13T 1S_Cd	14
T. carnifex	Bominaco	ZMA.RenA_7473	1C 14T 1S_Cd	15
T. carnifex	Bominaco	ZMA.RenA_7473	1C 14T 1S_Cd	15

T. carnifex	Bominaco	ZMA.RenA_7473	1C 14T 1S_Cd	15
T. carnifex	Farma	ZMA.RenA_8034	1C 13T 1S_Cd	14
T. carnifex	Farma	ZMA.RenA_8034	1C 13T 1S_Cd	14
T. carnifex	Haidlhof	ZMA.RenA_8051	1C 14T 1S_Cd	15
T. carnifex	Haidlhof	ZMA.RenA_8051	1C 13T 1S_Cd	14
T. carnifex	Geneve	ZMA.RenA_8074	1C 14T 1S_Cd	15
T. carnifex	Geneve	ZMA.RenA_8074	1C 13T 1S_Cd	14
T. carnifex	Geneve	ZMA.RenA_8074	1C 13T 1S_Cd	14
T. carnifex	Geneve	ZMA.RenA_8074	1C 13T 1S_Cd	14
T. carnifex	Geneve	ZMA.RenA_8074	1C 13T 1S_Cd	14
T. carnifex	Geneve	ZMA.RenA_8074	1C 13T 1S_Cd	14
T. carnifex	Geneve	ZMA.RenA_8074	1C 13T 1S_Cd	14
T. carnifex	Geneve	ZMA.RenA_8074	1C 13T 1S_Cd	14
T. carnifex	Geneve	ZMA.RenA_8074	1C 13T 1S_Cd	14
T. carnifex	Geneve	ZMA.RenA_8074	1C 13T 1S_Cd	14
T. carnifex	Sinac	ZMA.RenA_8082	1C 13T 1S_Cd	14
T. carnifex	Etzmannsdorf	ZMA.RenA_9105	1C 13T 1S_Cd	14
T. carnifex	Farma	ZMA.RenA_9106	1C 13T 1S_Cd	14
T. carnifex	Farma	ZMA.RenA_9106	1C 13T 1S_Cd	14
T. carnifex	Farma	ZMA.RenA_9106	1C 13T 1S_Cd	14
T. carnifex	Farma	ZMA.RenA_9106	1C 13T 1S_Cd	14
T. carnifex	Fuscaldo	ZMA.RenA_9108	1C 13T 1S_Cd	14
T. carnifex	Fuscaldo	ZMA.RenA_9108	1C 13T 1S_Cd	14
T. carnifex	Fuscaldo	ZMA.RenA_9108	1C 12T 1S_Cd	13
T. carnifex	Fuscaldo	ZMA.RenA_9108	1C 13T 1S_Cd	14
T. carnifex	Fuscaldo	ZMA.RenA_9108	1C 15T 1S_Cd	16
T. carnifex	Fuscaldo	ZMA.RenA_9108	1C 13T 1S_Cd	14
T. carnifex	Fuscaldo	ZMA.RenA_9108	1C 13T 1S_Cd	14
T. carnifex	Fuscaldo	ZMA.RenA_9108	1C 13T 1S_Cd	14
T. carnifex	Fuscaldo	ZMA.RenA_9108	1C 13T 1S_Cd	14
T. carnifex	Fuscaldo	ZMA.RenA_9108	1C 13T 1S_Cd	14
T. carnifex	Fuscaldo	ZMA.RenA_9108	1C 13T 1S_Cd	14
T. carnifex	Geneve	ZMA.RenA_9109	1C 14T 1S_Cd	15
T. carnifex	Geneve	ZMA.RenA_9109	1C 12T 1S_Cd	13

T. carnifex	Geneve	ZMA.RenA_9109	1C 13T 1S_Cd	14
T. carnifex	Geneve	ZMA.RenA_9109	1C 13T 1S_Cd	14
T. carnifex	Locarno	ZMA.RenA_9136	1C 13T 1S_Cd	14
T. carnifex	Locarno	ZMA.RenA_9136	1C 13T 1S_Cd	14
T. carnifex	Locarno	ZMA.RenA_9136	1C 12T 1S_Cd	13
T. carnifex	Locarno	ZMA.RenA_9136	1C 13T 1S_Cd	14
T. carnifex	Locarno	ZMA.RenA_9136	1C 13T 1S_Cd	14
T. carnifex	Pisa	ZMA.RenA_9145	1C 13T 1S_Cd	14
T. carnifex	Pisa	ZMA.RenA_9145	1C 13T 1S_Cd	14
T. carnifex	Pisa	ZMA.RenA_9145	1C 13T 1S_Cd	14
T. carnifex	Sinac	ZMA.RenA_9155	1C 12T 0.5T/S 0.5S/Cd_Cd	13.5
T. carnifex	Firenze	ZMA.RenA_9107	1C 13T 1S_Cd	14
T. carnifex	Firenze	ZMA.RenA_9107	1C 13T 1S_Cd	14
T. carnifex	Firenze	ZMA.RenA_9107	1C 13T 1S_Cd	14
T. carnifex	Firenze	ZMA.RenA_9107	1C 12T 0.5T/S 0.5S/Cd_Cd	13.5
T. carnifex	Firenze	ZMA.RenA_9107	1C 12T 0.5T/S 0.5S/Cd_Cd	13.5
T. carnifex	Firenze	ZMA.RenA_9107	1C 13T 1S_Cd	14
T. carnifex	Haidlhof	ZMA.RenA_9117	1C 13T 1S_Cd	14
T. carnifex	Haidlhof	ZMA.RenA_9117	1C 14T 1S_Cd	15
T. carnifex	Haidlhof	ZMA.RenA_9117	1C 13T 1S_Cd	14
T. carnifex	Haidlhof	ZMA.RenA_9117	1C 14T 1S_Cd	15
T. carnifex	Haidlhof	ZMA.RenA_9117	1C 13T 1S_Cd	14
T. carnifex	Bominaco	ZMA.RenA_ 7553	1C 13T 1S_Cd	14
T. carnifex	Bominaco	ZMA.RenA_ 7553	1C 13T 1S_Cd	14
T. carnifex	Fuscaldo	ZMA.RenA_ 7849	1C 13T 1S_Cd	14
T. carnifex	Geneva	ZMA.RenA_9110	1C 12T 1S_Cd	13
T. carnifex	Geneva	ZMA.RenA_9110	1C 13T 1S_Cd	14
T. carnifex	Geneva	ZMA.RenA_9110	1C 13T 1S_Cd	14
T. carnifex	Geneva	ZMA.RenA_9110	1C 14T 1S_Cd	15
T. carnifex	Geneva	ZMA.RenA_9110	1C 13T 1S_Cd	14
T. carnifex	Geneva	ZMA.RenA_9110	1C 13T 1S_Cd	14
T. carnifex	Geneva	ZMA.RenA_9110	1C 12T 1S_Cd	13
T. carnifex	Geneva	ZMA.RenA_9110	1C 13T 1S_Cd	14
T. carnifex	Geneva	ZMA.RenA_9110	1C 13T 1S_Cd	14

T. carnifex	Geneva	ZMA.RenA_9110	1C 13T 1S_Cd	14
T. carnifex	Geneva	ZMA.RenA_9110	1C 13T 1S_Cd	14
T. carnifex	Geneva	ZMA.RenA_9110	1C 13T 1S_Cd	14
T. carnifex	Geneva	ZMA.RenA_ 9110	1C 13T 1S_Cd	14
T. carnifex	Geneva	ZMA.RenA_ 9110	1C 13T 1S_Cd	14
T. carnifex	Geneva	ZMA.RenA_ 9110	1C 13T 1S_Cd	14
T. carnifex	Geneva	ZMA.RenA_ 9110	1C 13T 1S_Cd	14
T. carnifex	Geneva	ZMA.RenA_ 9110	1C 13T 1S_Cd	14
T. carnifex	Geneva	ZMA.RenA_ 9110	1C 12T 1S_Cd	13
T. carnifex	Geneva	ZMA.RenA_ 9110	1C 13T 1S_Cd	14
T. carnifex	Geneva	ZMA.RenA_ 9110	1C 13T 1S_Cd	14
T. carnifex	Geneva	ZMA.RenA_ 9110	1C 13T 1S_Cd	14
T. carnifex	Geneva	ZMA.RenA_ 9110	1C 13T 1S_Cd	14
T. carnifex	Geneva	ZMA.RenA_ 9110	1C 13T 1S_Cd	14
T. carnifex	Geneva	ZMA.RenA_ 9110	1C 13T 1S_Cd	14
T. carnifex	Klein-Meiseldorf	ZMA.RenA_9129	1C 14T 1S_Cd	15
T. carnifex	Klein-Meiseldorf	ZMA.RenA_9129	1C 14T 1S_Cd	15
T. carnifex	Klein-Meiseldorf	ZMA.RenA_9129	1C 13T 1S_Cd	14
T. carnifex	Klein-Meiseldorf	ZMA.RenA_9129	1C 13T 0.5T/S 0.5S/Cd_Cd	14.5
T. carnifex	Klein-Meiseldorf	ZMA.RenA_9129	1C 14T 1S_Cd	15
T. carnifex	Klein-Meiseldorf	ZMA.RenA_9129	1C 13T 0.5T/S 0.5S/Cd_Cd	14.5
T. carnifex	Klein-Meiseldorf	ZMA.RenA_9129	1C 14T 1S_Cd	15
T. carnifex	Klein-Meiseldorf	ZMA.RenA_9129	1C 14T 1S_Cd	15
T. carnifex	Klein-Meiseldorf	ZMA.RenA_9129	1C 14T 1S_Cd	15
T. carnifex	Klein-Meiseldorf	ZMA.RenA_9129	1C 13T 0.5T/S 0.5S/Cd_Cd	14.5
T. carnifex	Klein-Meiseldorf	ZMA.RenA_9129	1C 14T 1S_Cd	15
T. carnifex	Kramplje	ZMA.RenA_ 9132	1C 13T 1S_Cd	14
T. carnifex	Kramplje	ZMA.RenA_ 9132	1C 13T 1S_Cd	14
T. carnifex	Kramplje	ZMA.RenA_ 9132	1C 13T 1S_Cd	14
T. carnifex	Kramplje	ZMA.RenA_ 9132	1C 13T 1S_Cd	14
T. carnifex	Kramplje	ZMA.RenA_ 9132	1C 13T 1S_Cd	14
T. carnifex	Kramplje	ZMA.RenA_ 9132	1C 13T 1S_Cd	14
T. carnifex	Napoli	ZMA.RenA_ 9252	1C 12T 0.5T/S 0.5S/Cd_Cd	13.5
T. carnifex	Napoli	ZMA.RenA_ 9252	1C 12T 1S_Cd	13

T. cristatus	Bela Crkva	IBISS_20159	1C 15T 1S_Cd	16
T. cristatus	Bela Crkva	IBISS_20150	1C 14T 0.5T/S 0.5S/Cd_Cd	15.5
T. cristatus	Bela Crkva	IBISS_20156	1C 14T 0.5T/S 0.5S/Cd_Cd	15.5
T. cristatus	Bela Crkva	IBISS_20133	1C 14T 1S_Cd	15
T. cristatus	Bela Crkva	IBISS_20147	1C 14T 1S_Cd	15
T. cristatus	Bela Crkva	IBISS_20151	1C 14T 1S_Cd	15
T. cristatus	Bela Crkva	IBISS_20135	1C 14T 1S_Cd	15
T. cristatus	Bela Crkva	IBISS_20152	1C 14T 1S_Cd	15
T. cristatus	Bela Crkva	IBISS_20155	1C 14T 1S_Cd	15
T. cristatus	Bela Crkva	IBISS_20161	1C 14T 1S_Cd	15
T. cristatus	Bela Crkva	IBISS_20157	1C 14T 1S_Cd	15
T. cristatus	Bela Crkva	IBISS_20137	1C 14T 1S_Cd	15
T. cristatus	Bela Crkva	IBISS_20144	1C 14T 0.5T/S 0.5S/Cd_Cd	15.5
T. cristatus	Bela Crkva	IBISS_20160	1C 14T 1S_Cd	15
T. cristatus	Bela Crkva	IBISS_20158	1C 15T 1S_Cd	16
T. cristatus	Bela Crkva	IBISS_20153	1C 14T 1S_Cd	15
T. cristatus	Bela Crkva	IBISS_20141	1C 15T 1S_Cd	16
T. cristatus	Bela Crkva	IBISS_20154	1C 14T 1S_Cd	15
T. cristatus	Bela Crkva	IBISS_20148	0.5C/T 15.5T 1S_Cd	16
T. cristatus	Bela Crkva	IBISS_20142	1C 14T 1S_Cd	15
T. cristatus	Bela Crkva	IBISS_20143	1C 14T 1S_Cd	15
T. cristatus	Bela Crkva	IBISS_20134	1C 15T 1S_Cd	16
T. cristatus	Bela Crkva	IBISS_20138	1C 15T 1S_Cd	16
T. cristatus	Bela Crkva	IBISS_20140	1C 14T 1S_Cd	15
T. cristatus	Bela Crkva	IBISS_20163	1C 15T 1S_Cd	16
T. cristatus	Bela Crkva	IBISS_20136	1C 14T 1S_Cd	15
T. cristatus	Bela Crkva	IBISS_20145	1C 15T 1S_Cd	16
T. cristatus	Bela Crkva	IBISS_20162	1C 14T 1S_Cd	15
T. cristatus	Bela Crkva	IBISS_20131	1C 14T 1S_Cd	15
T. cristatus	Bela Crkva	IBISS_20132	1C 15T 1S_Cd	16
T. cristatus	Bela Crkva	IBISS_20149	1C 15T 1S_Cd	16
T. cristatus	Bela Crkva	IBISS_20139	1C 14T 1S_Cd	15
T. cristatus	Bela Crkva	IBISS_20146	1C 14T 1S_Cd	15
T. cristatus	Miroč	IBISS_20062	1C 13T 1S_Cd	14

T. cristatus	Miroč	IBISS_20060	1C 14T 1S_Cd	15
T. cristatus	Miroč	IBISS_20063	1C 14T 1S_Cd	15
T. cristatus	Miroč	IBISS_20061	1C 14T 1S_Cd	15
T. cristatus	Miroč	IBISS_20064	1C 14T 1S_Cd	15
T. cristatus	Miroč	IBISS_20047	1C 14T 1S_Cd	15
T. cristatus	Miroč	IBISS_20043	1C 14T 1S_Cd	15
T. cristatus	Miroč	IBISS_20045	1C 14T 1S_Cd	15
T. cristatus	Miroč	IBISS_20065	1C 14T 1S_Cd	15
T. cristatus	Miroč	IBISS_20058	1C 14T 1S_Cd	15
T. cristatus	Miroč	IBISS_20054	1C 14T 1S_Cd	15
T. cristatus	Miroč	IBISS_20052	1C 14T 1S_Cd	15
T. cristatus	Miroč	IBISS_20042	1C 14T 1S_Cd	15
T. cristatus	Miroč	IBISS_20049	1C 14T 1S_Cd	15
T. cristatus	Miroč	IBISS_20055	1C 14T 1S_Cd	15
T. cristatus	Miroč	IBISS_20079	1C 14T 1S_Cd	15
T. cristatus	Miroč	IBISS_20075	1C 14T 1S_Cd	15
T. cristatus	Miroč	IBISS_20070	1C 14T 1S_Cd	15
T. cristatus	Miroč	IBISS_20073	1C 13T 1S_Cd	14
T. cristatus	Miroč	IBISS_20072	1C 14T 1S_Cd	15
T. cristatus	Miroč	IBISS_20071	1C 14T 1S_Cd	15
T. cristatus	Miroč	IBISS_20080	1C 14T 1S_Cd	15
T. cristatus	Miroč	IBISS_20077	1C 14T 1S_Cd	15
T. cristatus	Miroč	IBISS_20074	1C 14T 1S_Cd	15
T. cristatus	Miroč	IBISS_20069	1C 14T 1S_Cd	15
T. cristatus	Miroč	IBISS_20057	1C 14T 1S_Cd	15
T. cristatus	Miroč	IBISS_20059	1C 13T 1S_Cd	14
T. cristatus	Miroč	IBISS_20056	1C 13T 1S_Cd	14
T. cristatus	Miroč	IBISS_20066	1C 14T 1S_Cd	15
T. cristatus	Miroč	IBISS_20050	1C 14T 1S_Cd	15
T. cristatus	Miroč	IBISS_20067	1C 14T 1S_Cd	15
T. cristatus	Miroč	IBISS_20068	1C 14T 1S_Cd	15
T. cristatus	Miroč	IBISS_20053	1C 14T 1S_Cd	15
T. cristatus	Miroč	IBISS_20051	1C 14T 1S_Cd	15
T. cristatus	Negotin	IBISS_6c27	1C 15T 1S_Cd	16

T. cristatus	Negotin	IBISS_13c27	1C 13T 1S_Cd	14
T. cristatus	Negotin	IBISS_2c27	1C 14T 1S_Cd	15
T. cristatus	Negotin	IBISS_7c27	1C 14T 1S_Cd	15
T. cristatus	Negotin	IBISS_9c27	1C 14T 1S_Cd	15
T. cristatus	Negotin	IBISS_5c27	1C 14T 1S_Cd	15
T. cristatus	Negotin	IBISS_12c27	1C 14T 1S_Cd	15
T. cristatus	Negotin	IBISS_3c27	1C 14T 1S_Cd	15
T. cristatus	Negotin	IBISS_11c27	1C 15T 1S_Cd	16
T. cristatus	Negotin	IBISS_1c27	1C 14T 1S_Cd	15
T. cristatus	Negotin	IBISS_7c27	1C 14T 1S_Cd	15
T. cristatus	Negotin	IBISS_11c27	1C 14T 1S_Cd	15
T. cristatus	Negotin	IBISS_13c27	1C 14T 1S_Cd	15
T. cristatus	Negotin	IBISS_8c27	1C 14T 1S_Cd	15
T. cristatus	Negotin	IBISS_15c27	1C 14T 1S_Cd	15
T. cristatus	Negotin	IBISS_10c27	1C 14T 1S_Cd	15
T. cristatus	Negotin	IBISS_3c27	1C 14T 1S_Cd	15
T. cristatus	Negotin	IBISS_4c27	1C 14T 1S_Cd	15
T. cristatus	Negotin	IBISS_16c27	1C 14T 1S_Cd	15
T. cristatus	Negotin	IBISS_1c27	1C 14T 1S_Cd	15
T. cristatus	Mayenne	ZMA.RenA_7420	1C 14T 1S_Cd	15
T. cristatus	Mayenne	ZMA.RenA_7420	1C 14T 1S_Cd	15
T. cristatus	Mayenne	ZMA.RenA_7420	1C 14T 1S_Cd	15
T. cristatus	Mayenne	ZMA.RenA_7420	1C 14T 1S_Cd	15
T. cristatus	Mayenne	ZMA.RenA_7420	1C 14T 1S_Cd	15
T. cristatus	Mayenne	ZMA.RenA_7420	1C 14T 1S_Cd	15
T. cristatus	Mayenne	ZMA.RenA_7420	1C 14T 1S_Cd	15
T. cristatus	Mayenne	ZMA.RenA_7420	1C 14T 1S_Cd	15
T. cristatus	Mayenne	ZMA.RenA_7421	1C 14T 1S_Cd	15
T. cristatus	Mayenne	ZMA.RenA_7421	1C 14T 1S_Cd	15
T. cristatus	Mayenne	ZMA.RenA_7421	1C 14T 1S_Cd	15
T. cristatus	Mayenne	ZMA.RenA_7421	1C 14T 1S_Cd	15
T. cristatus	Mayenne	ZMA.RenA_7421	1C 14T 1S_Cd	15
T. cristatus	Mayenne	ZMA.RenA_7421	1C 14T 1S_Cd	15
T. cristatus	Mayenne	ZMA.RenA_7421	1C 14T 1S_Cd	15

T. cristatus	Mayenne	ZMA.RenA_7421	1C 14T 1S_Cd	15
T. cristatus	Mayenne	ZMA.RenA_7421	1C 13T 1S_Cd	14
T. cristatus	Mayenne	ZMA.RenA_7421	1C 14T 1S_Cd	15
T. cristatus	Mayenne	ZMA.RenA_7421	1C 13T 1S_Cd	14
T. cristatus	Mayenne	ZMA.RenA_7421	1C 14T 1S_Cd	15
T. cristatus	Mayenne	ZMA.RenA_7421	1C 14T 1S_Cd	15
T. cristatus	Mayenne	ZMA.RenA_7421	1C 14T 0.5T/S 0.5S/Cd_Cd	15.5
T. cristatus	Mayenne	ZMA.RenA_7421	1C 14T 1S_Cd	15
T. cristatus	Mayenne	ZMA.RenA_7421	1C 14T 1S_Cd	15
T. cristatus	Mayenne	ZMA.RenA_7421	1C 14T 1S_Cd	15
T. cristatus	Mayenne	ZMA.RenA_7421	1C 14T 0.5T/S 0.5S/Cd_Cd	15.5
T. cristatus	Mayenne	ZMA.RenA_7421	1C 14T 1S_Cd	15
T. cristatus	Mayenne	ZMA.RenA_7549	1C 14T 1S_Cd	15
T. cristatus	Mayenne	ZMA.RenA_7549	1C 14T 1S_Cd	15
T. cristatus	Mayenne	ZMA.RenA_7549	1C 14T 1S_Cd	15
T. cristatus	Mayenne	ZMA.RenA_7549	1C 14T 1S_Cd	15
T. cristatus	Mayenne	ZMA.RenA_7549	1C 14T 1S_Cd	15
T. cristatus	Mayenne	ZMA.RenA_7549	1C 15T 1S_Cd	16
T. cristatus	Lanckorona	ZMA.RenA_7581	1C 14T 1S_Cd	15
T. cristatus	Lanckorona	ZMA.RenA_7581	1C 14T 1S_Cd	15
T. cristatus	Lanckorona	ZMA.RenA_7581	1C 14T 1S_Cd	15
T. cristatus	Lanckorona	ZMA.RenA_7581	1C 14T 1S_Cd	15
T. cristatus	Lanckorona	ZMA.RenA_7581	1C 14T 1S_Cd	15
T. cristatus	Lanckorona	ZMA.RenA_7581	1C 14T 1S_Cd	15
T. cristatus	Lanckorona	ZMA.RenA_7581	1C 13T 1S_Cd	14
T. cristatus	Lanckorona	ZMA.RenA_7581	1C 14T 1S_Cd	15
T. cristatus	Ambleteuse	ZMA.RenA_7619	1C 14T 0.5T/S 0.5S/Cd_Cd	15.5
T. cristatus	Ambleteuse	ZMA.RenA_7619	1C 14T 1S_Cd	15
T. cristatus	Bor	ZMA.RenA_7848	1C 14T 1S_Cd	15
T. cristatus	Mayenne	ZMA.RenA_7991	1C 14T 1S_Cd	15
T. cristatus	Mayenne	ZMA.RenA_7991	1C 14T 1S_Cd	15
T. cristatus	Sebis	ZMA.RenA_8055	1C 14T 1S_Cd	15
T. cristatus	Sebis	ZMA.RenA_8055	1C 14T 1S_Cd	15
T. cristatus	Sebis	ZMA.RenA_8055	1C 14T 1S_Cd	15

T. cristatus	Mayenne	ZMA.RenA_8090	1C 14T 1S_Cd	15
T. cristatus	Mayenne	ZMA.RenA_8090	1C 14T 1S_Cd	15
T. cristatus	Mayenne	ZMA.RenA_8090	1C 14T 1S_Cd	15
T. cristatus	Mayenne	ZMA.RenA_8090	1C 14T 1S_Cd	15
T. cristatus	Mayenne	ZMA.RenA_8090	1C 14T 1S_Cd	15
T. cristatus	Mayenne	ZMA.RenA_8090	1C 15T 1S_Cd	16
T. cristatus	Mayenne	ZMA.RenA_8090	1C 14T 1S_Cd	15
T. cristatus	Mayenne	ZMA.RenA_8090	1C 15T 1S_Cd	16
T. cristatus	Mayenne	ZMA.RenA_8090	1C 13T 1S_Cd	14
T. cristatus	Mayenne	ZMA.RenA_9068	1C 14T 0.5T/S 0.5S/Cd_Cd	15.5
T. cristatus	Mayenne	ZMA.RenA_9069	1C 14T 1S_Cd	15
T. cristatus	Mayenne	ZMA.RenA_9069	1C 14T 1S_Cd	15
T. cristatus	Mayenne	ZMA.RenA_9069	1C 14T 1S_Cd	15
T. cristatus	Mayenne	ZMA.RenA_9069	1C 14T 1S_Cd	15
T. cristatus	Mayenne	ZMA.RenA_9069	1C 14T 1S_Cd	15
T. cristatus	Mayenne	ZMA.RenA_9071	1C 14T 1S_Cd	15
T. cristatus	Biel	ZMA.RenA_9071	1C 14T 1S_Cd	15
T. cristatus	Bor	ZMA.RenA_9093	1C 14T 1S_Cd	15
T. cristatus	Bor	ZMA.RenA_9093	1C 14T 1S_Cd	15
T. cristatus	Bor	ZMA.RenA_9093	1C 14T 1S_Cd	15
T. cristatus	Kladovo	ZMA.RenA_9128	1C 16T 1S_Cd	17
T. cristatus	Kladovo	ZMA.RenA_9128	1C 15T 1S_Cd	16
T. cristatus	Klokočevac	ZMA.RenA_9130	1C 13T 1S_Cd	14
T. cristatus	Klokočevac	ZMA.RenA_9130	1C 14T 1S_Cd	15
T. cristatus	Klokočevac	ZMA.RenA_9130	1C 13T 1S_Cd	14
T. cristatus	Klokočevac	ZMA.RenA_9130	1C 14T 1S_Cd	15
T. cristatus	Ottenstein	ZMA.RenA_9143	1C 14T 1S_Cd	15
T. cristatus	Sebis	ZMA.RenA_9152	1C 14T 1S_Cd	15
T. cristatus	Stubik	ZMA.RenA_9159	1C 14T 1S_Cd	15
T. cristatus	Stubik	ZMA.RenA_9159	1C 14T 1S_Cd	15
T. cristatus	Stubik	ZMA.RenA_9159	1C 14T 1S_Cd	15
T. cristatus	Stubik	ZMA.RenA_9159	1C 14T 1S_Cd	15
T. cristatus	Tirgovište	ZMA.RenA_9163	1C 14T 1S_Cd	15
T. cristatus	Tirgovište	ZMA.RenA_9163	1C 13T 0.5T/S 0.5S/Cd_Cd	14.5

T. cristatus	Tirgovište	ZMA.RenA_9163	1C 14T 1S_Cd	15
T. cristatus	Tirgovište	ZMA.RenA_9163	1C 14T 1S_Cd	15
T. cristatus	Tirgovište	ZMA.RenA_9163	1C 14T 1S_Cd	15
T. cristatus	Tirgovište	ZMA.RenA_9163	1C 14T 1S_Cd	15
T. cristatus	Tirgovište	ZMA.RenA_9163	1C 14T 1S_Cd	15
T. cristatus	Tirgovište	ZMA.RenA_9163	1C 14T 1S_Cd	15
T. cristatus	Virfuri	ZMA.RenA_9167	1C 14T 1S_Cd	15
T. cristatus	Virfuri	ZMA.RenA_9167	1C 14T 1S_Cd	15
T. cristatus	Virfuri	ZMA.RenA_9167	1C 14T 1S_Cd	15
T. cristatus	Virfuri	ZMA.RenA_9167	1C 14T 1S_Cd	15
T. cristatus	Virfuri	ZMA.RenA_9167	1C 14T 1S_Cd	15
T. cristatus	Virfuri	ZMA.RenA_9167	1C 14T 1S_Cd	15
T. cristatus	Virfuri	ZMA.RenA_9167	1C 14T 1S_Cd	15
T. cristatus	Virfuri	ZMA.RenA_9167	1C 14T 1S_Cd	15
T. cristatus	Virfuri	ZMA.RenA_9167	1C 14T 1S_Cd	15
T. cristatus	Virfuri	ZMA.RenA_9167	1C 16T 1S_Cd	17
T. cristatus	Mayenne	ZMA.RenA_9263	1C 14T 1S_Cd	15
T. cristatus	Mayenne	ZMA.RenA_9263	1C 14T 1S_Cd	15
T. cristatus	Mayenne	ZMA.RenA_9264	1C 14T 1S_Cd	15
T. cristatus	Mayenne	ZMA.RenA_9264	1C 15T 1S_Cd	16
T. cristatus	Braila	ZMA.RenA_9304	1C 15T 1S_Cd	16
T. cristatus	Jabukovac	ZMA.RenA_9119	1C 14T 1S_Cd	15
T. cristatus	Jabukovac	ZMA.RenA_9119	1C 14T 1S_Cd	15
T. cristatus	Jabukovac	ZMA.RenA_9119	1C 14T 1S_Cd	15
T. cristatus	Jabukovac	ZMA.RenA_9119	1C 14T 1S_Cd	15
T. cristatus	Jabukovac	ZMA.RenA_9119	1C 14T 1S_Cd	15
T. cristatus	Lukovo	ZMA.RenA_9138	1C 12T 1S_Cd	13
T. cristatus	Peterborough	ZMA.RenA_9144	1C 15T 1S_Cd	16
T. cristatus	Sinaia	ZMA.RenA_9156	1C 13T 1S_Cd	14
T. cristatus	Sinaia	ZMA.RenA_9156	1C 14T 1S_Cd	15
T. cristatus	Sinaia	ZMA.RenA_9156	1C 14T 1S_Cd	15
T. cristatus	Sinaia	ZMA.RenA_9156	1C 14T 1S_Cd	15
T. cristatus	Sinaia	ZMA.RenA_9156	1C 14T 1S_Cd	15
T. cristatus	Sinaia	ZMA.RenA_9156	1C 14T 1S_Cd	15

T. cristatus	Sinaia	ZMA.RenA_9156	1C 14T 1S_Cd	15
T. cristatus	Sinaia	ZMA.RenA_9156	1C 13T 0.5T/S 0.5S/Cd_Cd	14.5
T. cristatus	Sinaia	ZMA.RenA_9156	1C 14T 1S_Cd	15
T. cristatus	Sinaia	ZMA.RenA_9156	1C 14T 1S_Cd	15
T. cristatus	Peterborough	ZMA.RenA_9199	1C 14T 1S_Cd	15
T. cristatus	Peterborough	ZMA.RenA_9199	1C 14T 1S_Cd	15
T. cristatus	Peterborough	ZMA.RenA_9199	1C 14T 1S_Cd	15
T. cristatus	Peterborough	ZMA.RenA_9199	1C 14T 1S_Cd	15
T. cristatus	Tirgoviste	ZMA.RenA_9163	1C 14T 1S_Cd	15
T. cristatus	Videle	ZMA.RenA_9166	1C 14T 1S_Cd	15
T. cristatus	Videle	ZMA.RenA_9166	0C 15T 1S_Cd	15
T. cristatus	Videle	ZMA.RenA_9166	1C 14T 1S_Cd	15
T. cristatus	Videle	ZMA.RenA_9166	1C 14T 1S_Cd	15
T. cristatus	Videle	ZMA.RenA_9166	1C 14T 1S_Cd	15
T. cristatus	Videle	ZMA.RenA_9166	1C 13T 1S_Cd	14
T. cristatus	Videle	ZMA.RenA_9166	1C 14T 1S_Cd	15
T. cristatus	Videle	ZMA.RenA_9166	1C 13T 1S_Cd	14
T. cristatus	Videle	ZMA.RenA_9166	1C 14T 1S_Cd	15
T. cristatus	Videle	ZMA.RenA_9166	1C 14T 1S_Cd	15
T. cristatus	Canterbury	ZMA.RenA_9200	1C 14T 1S_Cd	15
T. cristatus	Canterbury	ZMA.RenA_9200	1C 14T 1S_Cd	15
T. cristatus	Canterbury	ZMA.RenA_9200	1C 13T 1S_Cd	14
T. cristatus	Saint-Lô	ZMA.RenA_9265	1C 14T 1S_Cd	15
T. cristatus	Mayenne	ZMA.RenA_9240	1C 15T 1S_Cd	16
T. cristatus	Mayenne	ZMA.RenA_9240	1C 14T 1S_Cd	15
T. cristatus	Mayenne	ZMA.RenA_9240	1C 14T 1S_Cd	15
T. cristatus	Mayenne	ZMA.RenA_ 8408	1C 14T 1S_Cd	15
T. cristatus	Mayenne	ZMA.RenA_8409	1C 15T 1S_Cd	16
T. cristatus	Mayenne	ZMA.RenA_ 8410	1C 14T 1S_Cd	15
T. cristatus	Biel	ZMA.RenA_ 8056	1C 15T 1S_Cd	16
T. cristatus	Biel	ZMA.RenA_ 8056	1C 15T 1S_Cd	16
T. cristatus	Biel	ZMA.RenA_ 8056	1C 14T 1S_Cd	15
T. cristatus	Biel	ZMA.RenA_ 8056	1C 14T 1S_Cd	15
T. cristatus	Mayenne	ZMA.RenA_ 9070	1C 14T 1S_Cd	15

T. cristatus	Mayenne	ZMA.RenA_ 9070	1C 13T 0.5T/S 0.5S/Cd_Cd	14.5
T. cristatus	Mayenne	ZMA.RenA_ 9070	1C 14T 1S_Cd	15
T. cristatus	Mayenne	ZMA.RenA_ 9070	1C 14T 1S_Cd	15
T. cristatus	Mayenne	ZMA.RenA_ 9070	1C 14T 1S_Cd	15
T. cristatus	Mayenne	ZMA.RenA_ 9070	1C 14T 1S_Cd	15
T. cristatus	Mayenne	ZMA.RenA_ 9070	1C 14T 1S_Cd	15
T. cristatus	Mayenne	ZMA.RenA_ 9070	1C 14T 1S_Cd	15
T. cristatus	Mayenne	ZMA.RenA_ 9070	1C 13T 1S_Cd	14
T. cristatus	Mayenne	ZMA.RenA_ 9070	1C 14T 0.5T/S 0.5S/Cd_Cd	15.5
T. cristatus	Mayenne	ZMA.RenA_ 9070	1C 14T 1S_Cd	15
T. cristatus	Mayenne	ZMA.RenA_ 9070	1C 14T 1S_Cd	15
T. cristatus	Mayenne	ZMA.RenA_ 9070	1C 14T 1S_Cd	15
T. cristatus	Mayenne	ZMA.RenA_ 9070	1C 14T 1S_Cd	15
T. cristatus	Mayenne	ZMA.RenA_ 9070	1C 14T 1S_Cd	15
T. cristatus	Mayenne	ZMA.RenA_ 9070	1C 14T 1S_Cd	15
T. cristatus	Mayenne	ZMA.RenA_9072	1C 14T 1S_Cd	15
T. cristatus	Mayenne	ZMA.RenA_9072	1C 14T 1S_Cd	15
T. cristatus	Mayenne	ZMA.RenA_9072	1C 14T 1S_Cd	15
T. cristatus	Mayenne	ZMA.RenA_9072	1C 14T 1S_Cd	15
T. cristatus	Mayenne	ZMA.RenA_9072	1C 15T 1S_Cd	16
T. cristatus	Mayenne	ZMA.RenA_9072	1C 14T 1S_Cd	15
T. cristatus	Mayenne	ZMA.RenA_9072	1C 14T 1S_Cd	15
T. cristatus	Mayenne	ZMA.RenA_9072	1C 12T 1S_Cd	13
T. cristatus	Mayenne	ZMA.RenA_9072	1C 14T 1S_Cd	15
T. cristatus	Mayenne	ZMA.RenA_9072	1C 14T 1S_Cd	15
T. cristatus	Mayenne	ZMA.RenA_9073	1C 14T 1S_Cd	15
T. cristatus	Campeni	ZMA.RenA_9094	1C 13T 0.5T/S 0.5S/Cd_Cd	14.5
T. cristatus	Campeni	ZMA.RenA_9094	1C 14T 1S_Cd	15
T. cristatus	Campeni	ZMA.RenA_9094	1C 14T 1S_Cd	15
T. cristatus	Campeni	ZMA.RenA_9094	1C 14T 1S_Cd	15
T. cristatus	Campeni	ZMA.RenA_9094	1C 14T 1S_Cd	15
T. cristatus	Campeni	ZMA.RenA_9094	1C 14T 1S_Cd	15
T. cristatus	Campeni	ZMA.RenA_9094	1C 14T 1S_Cd	15
T. cristatus	Campeni	ZMA.RenA_9094	1C 14T 1S_Cd	15

T. cristatus	Campeni	ZMA.RenA_9094	1C 14T 1S_Cd	15
T. cristatus	Campeni	ZMA.RenA_9094	1C 14T 1S_Cd	15
T. cristatus	Limanowa	ZMA.RenA_ 9135	1C 14T 1S_Cd	15
T. cristatus	Limanowa	ZMA.RenA_ 9135	1C 14T 1S_Cd	15
T. cristatus	Limanowa	ZMA.RenA_ 9135	1C 14T 1S_Cd	15
T. cristatus	Limanowa	ZMA.RenA_ 9135	1C 14T 1S_Cd	15
T. cristatus	Limanowa	ZMA.RenA_ 9135	1C 14T 1S_Cd	15
T. cristatus	Milanovac	ZMA.RenA_9139	1C 14T 1S_Cd	15
T. cristatus	Milanovac	ZMA.RenA_9139	0C 15T 1S_Cd	15
T. cristatus	Milanovac	ZMA.RenA_9139	1C 13T 1S_Cd	14
T. cristatus	Milanovac	ZMA.RenA_9139	1C 13T 0.5T/S 0.5S/Cd_Cd	14.5
T. cristatus	Milanovac	ZMA.RenA_9139	1C 14T 1S_Cd	15
T. cristatus	Milanovac	ZMA.RenA_9139	1C 14T 1S_Cd	15
T. cristatus	Milanovac	ZMA.RenA_9139	1C 14T 1S_Cd	15
T. dobrogicus	Ivanovo	IBISS_G22550	1C 15T 1S_Cd	16
T. dobrogicus	Ivanovo	IBISS_G22552	1C 16T 1S_Cd	17
T. dobrogicus	Ivanovo	IBISS_G22555	1C 15T 1S_Cd	16
T. dobrogicus	Ivanovo	IBISS_G22556	1C 15T 1S_Cd	16
T. dobrogicus	Ivanovo	IBISS_G22558	1C 15T 1S_Cd	16
T. dobrogicus	Ivanovo	IBISS_G22540	1C 15T 1S_Cd	16
T. dobrogicus	Ivanovo	IBISS_G22557	1C 15T 1S_Cd	16
T. dobrogicus	Ivanovo	IBISS_G22547	1C 15T 1S_Cd	16
T. dobrogicus	Ivanovo	IBISS_G22559	1C 16T 1S_Cd	17
T. dobrogicus	Ivanovo	IBISS_G22554	1C 15T 1S_Cd	16
T. dobrogicus	Ivanovo	IBISS_G22549	1C 15T 0.5T/S 0.5S/Cd_Cd	16.5
T. dobrogicus	Ivanovo	IBISS_G22546	1C 17T 1S_Cd	18
T. dobrogicus	Ivanovo	IBISS_G22544	1C 16T 1S_Cd	17
T. dobrogicus	Ivanovo	IBISS_G22551	1C 16T 1S_Cd	17
T. dobrogicus	Ivanovo	IBISS_G22543	1C 15T 1S_Cd	16
T. dobrogicus	Ivanovo	IBISS_G22542	1C 16T 1S_Cd	17
T. dobrogicus	Ivanovo	IBISS_G22545	1C 16T 1S_Cd	17
T. dobrogicus	Ivanovo	IBISS_G22541	1C 16T 1S_Cd	17
T. dobrogicus	Ivanovo	IBISS_G22553	1C 15T 1S_Cd	16
T. dobrogicus	Ivanovo	IBISS_G22548	1C 15T 1S_Cd	16

T. dobrogicus	Ivanovo	IBISS_15c10	1C 16T 1S_Cd	17
T. dobrogicus	Ivanovo	IBISS_16c10	1C 15T 1S_Cd	16
T. dobrogicus	Ivanovo	IBISS_18c10	1C 16T 1S_Cd	17
T. dobrogicus	Ivanovo	IBISS_9c10	1C 16T 1S_Cd	17
T. dobrogicus	Ivanovo	IBISS_3c10	1C 15T 1S_Cd	16
T. dobrogicus	Ivanovo	IBISS_2c10	1C 16T 1S_Cd	17
T. dobrogicus	Ivanovo	IBISS_1c10	1C 16T 1S_Cd	17
T. dobrogicus	Ivanovo	IBISS_17c10	1C 16T 0.5T/S 0.5S/Cd_Cd	17.5
T. dobrogicus	Ivanovo	IBISS_14c10	1C 16T 1S_Cd	17
T. dobrogicus	Ivanovo	IBISS_14c10	1C 15T 1S_Cd	16
T. dobrogicus	Ivanovo	IBISS_8c10	1C 16T 1S_Cd	17
T. dobrogicus	Ivanovo	IBISS_11c10	1C 16T 1S_Cd	17
T. dobrogicus	Ivanovo	IBISS_5c10	1C 16T 1S_Cd	17
T. dobrogicus	Ivanovo	IBISS_6c10	1C 15T 1S_Cd	16
T. dobrogicus	Ivanovo	IBISS_7c10	1C 16T 1S_Cd	17
T. dobrogicus	Ivanovo	IBISS_30c10	1C 15T 1S_Cd	16
T. dobrogicus	Ivanovo	IBISS_20c10	1C 15T 1S_Cd	16
T. dobrogicus	Ivanovo	IBISS_16c10	1C 16T 1S_Cd	17
T. dobrogicus	Ivanovo	IBISS_6c10	1C 16T 1S_Cd	17
T. dobrogicus	Ivanovo	IBISS_13c10	1C 15T 0.5T/S 0.5S/Cd_Cd	16.5
T. dobrogicus	Ivanovo	IBISS_24c10	1C 16T 1S_Cd	17
T. dobrogicus	Ivanovo	IBISS_11c10	1C 16T 1S_Cd	17
T. dobrogicus	Ivanovo	IBISS_10c10	1C 16T 1S_Cd	17
T. dobrogicus	Ivanovo	IBISS_2c10	1C 16T 1S_Cd	17
T. dobrogicus	Ivanovo	IBISS_23c10	1C 16T 1S_Cd	17
T. dobrogicus	Ivanovo	IBISS_3c10	1C 16T 1S_Cd	17
T. dobrogicus	Ivanovo	IBISS_1c10	1C 15T 1S_Cd	16
T. dobrogicus	Ivanovo	IBISS_17c10	1C 15T 1S_Cd	16
T. dobrogicus	Ivanovo	IBISS_12c10	1C 16T 1S_Cd	17
T. dobrogicus	Ivanovo	IBISS_4c10	1C 16T 1S_Cd	17
T. dobrogicus	Ivanovo	IBISS_4c10	1C 15T 0.5T/S 0.5S/Cd_Cd	16.5
T. dobrogicus	Opovo	IBISS_G22924	1C 16T 1S_Cd	17
T. dobrogicus	Opovo	IBISS_G22925	1C 16T 1S_Cd	17
T. dobrogicus	Opovo	IBISS_G22928	1C 15T 0.5T/S 0.5S/Cd_Cd	16.5

T. dobrogicus	Opovo	IBISS_G22929	1C 15T 1S_Cd	16
T. dobrogicus	Opovo	IBISS_G22930	1C 16T 1S_Cd	17
T. dobrogicus	Opovo	IBISS_G22931	1C 16T 1S_Cd	17
T. dobrogicus	Opovo	IBISS_G22932	1C 15T 1S_Cd	16
T. dobrogicus	Opovo	IBISS_G22933	1C 16T 1S_Cd	17
T. dobrogicus	Opovo	IBISS_G22934	1C 15T 1S_Cd	16
T. dobrogicus	Opovo	IBISS_G22935	1C 15T 0.5T/S 0.5S/Cd_Cd	16.5
T. dobrogicus	Opovo	IBISS_G22936	1C 15T 1S_Cd	16
T. dobrogicus	Opovo	IBISS_G22937	1C 15T 1S_Cd	16
T. dobrogicus	Kikinda	IBISS_G22938	1C 16T 1S_Cd	17
T. dobrogicus	Kikinda	IBISS_G22939	1C 16T 1S_Cd	17
T. dobrogicus	Kikinda	IBISS_G22940	1C 15T 1S_Cd	16
T. dobrogicus	Kikinda	IBISS_G22941	1C 16T 1S_Cd	17
T. dobrogicus	Kikinda	IBISS_G22942	1C 16T 1S_Cd	17
T. dobrogicus	Kikinda	IBISS_G22943	1C 16T 1S_Cd	17
T. dobrogicus	Kikinda	IBISS_G22944	1C 16T 1S_Cd	17
T. dobrogicus	Kikinda	IBISS_G22945	1C 16T 1S_Cd	17
T. dobrogicus	Kikinda	IBISS_G22946	1C 16T 1S_Cd	17
T. dobrogicus	Marchegg	ZMA.RenA_7640	1C 16T 1S_Cd	17
T. dobrogicus	Öcsöd	ZMA.RenA_8040	1C 16T 1S_Cd	17
T. dobrogicus	Alap	ZMA.RenA_8067	1C 15T 1S_Cd	16
T. dobrogicus	Alap	ZMA.RenA_8067	1C 15T 1S_Cd	16
T. dobrogicus	Alap	ZMA.RenA_8067	1C 15T 1S_Cd	16
T. dobrogicus	Ečka	ZMA.RenA_8068	1C 15T 1S_Cd	16
T. dobrogicus	Ečka	ZMA.RenA_8068	1C 14T 1S_Cd	15
T. dobrogicus	Opovo	ZMA.RenA_8069	1C 14T 1S_Cd	15
T. dobrogicus	Senta	ZMA.RenA_8070	1C 16T 1S_Cd	17
T. dobrogicus	Öcsöd	ZMA.RenA_8071	1C 17T 1S_Cd	18
T. dobrogicus	Donja Čađavica	ZMA.RenA_8077	1C 14T 1S_Cd	15
T. dobrogicus	Donja Čađavica	ZMA.RenA_8077	1C 14T 1S_Cd	15
T. dobrogicus	Donja Čađavica	ZMA.RenA_8077	1C 14T 1S_Cd	15
T. dobrogicus	Jamena	ZMA.RenA_8084	1C 16T 1S_Cd	17
T. dobrogicus	Donja Čađavica	ZMA.RenA_9101	1C 13T 0.5T/S 0.5S/Cd_Cd	14.5
T. dobrogicus	Donja Čađavica	ZMA.RenA_9101	1C 15T 1S_Cd	16

T. dobrogicus	Donja Čađavica	ZMA.RenA_9101	1C 14T 1S_Cd	15
T. dobrogicus	Donja Čađavica	ZMA.RenA_9101	1C 15T 1S_Cd	16
T. dobrogicus	Donja Čađavica	ZMA.RenA_9101	1C 14T 1S_Cd	15
T. dobrogicus	Donja Čađavica	ZMA.RenA_9101	1C 13T 1S_Cd	14
T. dobrogicus	Donja Čađavica	ZMA.RenA_9101	1C 14T 1S_Cd	15
T. dobrogicus	Donja Čađavica	ZMA.RenA_9101	1C 15T 1S_Cd	16
T. dobrogicus	Dugo Selo	ZMA.RenA_9103	1C 16T 1S_Cd	17
T. dobrogicus	Körmend	ZMA.RenA_9131	1C 16T 1S_Cd	17
T. dobrogicus	Öcsöd	ZMA.RenA_9141	1C 16T 1S_Cd	17
T. dobrogicus	Öcsöd	ZMA.RenA_9141	1C 16T 1S_Cd	17
T. dobrogicus	Öcsöd	ZMA.RenA_9141	1C 16T 1S_Cd	17
T. dobrogicus	Öcsöd	ZMA.RenA_9141	1C 16T 1S_Cd	17
T. dobrogicus	Öcsöd	ZMA.RenA_9141	1C 16T 1S_Cd	17
T. dobrogicus	Öcsöd	ZMA.RenA_9141	1C 16T 1S_Cd	17
T. dobrogicus	Öcsöd	ZMA.RenA_9141	1C 16T 1S_Cd	17
T. dobrogicus	Sebis	ZMA.RenA_9152	1C 16T 1S_Cd	17
T. dobrogicus	Sebis	ZMA.RenA_9152	1C 15T 1S_Cd	16
T. dobrogicus	Sebis	ZMA.RenA_9152	1C 16T 1S_Cd	17
T. dobrogicus	Sebis	ZMA.RenA_9152	1C 14T 1S_Cd	15
T. dobrogicus	Sebis	ZMA.RenA_9152	1C 14T 1S_Cd	15
T. dobrogicus	Svistov	ZMA.RenA_9160	1C 16T 1S_Cd	17
T. dobrogicus	Svistov	ZMA.RenA_9160	1C 16T 1S_Cd	17
T. dobrogicus	Szolnok	ZMA.RenA_9161	1C 16T 1S_Cd	17
T. dobrogicus	Zupanja	ZMA.RenA_9172	1C 15T 1S_Cd	16
T. dobrogicus	Zupanja	ZMA.RenA_9172	1C 15T 1S_Cd	16
T. dobrogicus	Zupanja	ZMA.RenA_9172	1C 15T 1S_Cd	16
T. dobrogicus	Zupanja	ZMA.RenA_9172	1C 15T 1S_Cd	16
T. dobrogicus	Zupanja	ZMA.RenA_9172	1C 15T 1S_Cd	16
T. dobrogicus	Zupanja	ZMA.RenA_9172	1C 15T 1S_Cd	16
T. dobrogicus	Debrc	ZMA.RenA_9097	1C 15T 1S_Cd	16
T. dobrogicus	Debrc	ZMA.RenA_9097	1C 15T 1S_Cd	16
T. dobrogicus	Debrc	ZMA.RenA_9097	1C 15T 1S_Cd	16
T. dobrogicus	Debrc	ZMA.RenA_9097	1C 15T 1S_Cd	16
T. dobrogicus	Debrc	ZMA.RenA_9097	1C 16T 1S_Cd	17

T. dobrogicus	Debrc	ZMA.RenA_9097	1C 16T 1S_Cd	17
T. dobrogicus	Debrc	ZMA.RenA_9097	1C 15T 1S_Cd	16
T. dobrogicus	Debrc	ZMA.RenA_9097	1C 16T 1S_Cd	17
T. dobrogicus	Debrc	ZMA.RenA_9097	1C 15T 1S_Cd	16
T. dobrogicus	Debrc	ZMA.RenA_9097	1C 15T 1S_Cd	16
T. dobrogicus	Debrc	ZMA.RenA_9097	1C 15T 1S_Cd	16
T. dobrogicus	Debrc	ZMA.RenA_9097	1C 15T 1S_Cd	16
T. dobrogicus	Debrc	ZMA.RenA_9097	1C 16T 1S_Cd	17
T. dobrogicus	Debrc	ZMA.RenA_9097	1C 15T 0.5T/S 0.5S/Cd_Cd	16.5
T. dobrogicus	Debrc	ZMA.RenA_9097	1C 15T 0.5T/S 0.5S/Cd_Cd	16.5
T. dobrogicus	Debrc	ZMA.RenA_9097	1C 16T 1S_Cd	17
T. dobrogicus	Debrc	ZMA.RenA_9097	1C 15T 1S_Cd	16
T. dobrogicus	Debrc	ZMA.RenA_9097	1C 16T 1S_Cd	17
T. dobrogicus	Debrc	ZMA.RenA_9097	1C 16T 1S_Cd	17
T. dobrogicus	Debrc	ZMA.RenA_9097	1C 15T 0.5T/S 0.5S/Cd_Cd	16.5
T. dobrogicus	Debrc	ZMA.RenA_9097	1C 15T 1S_Cd	16
T. dobrogicus	Debrc	ZMA.RenA_9097	1C 15T 1S_Cd	16
T. dobrogicus	Debrc	ZMA.RenA_9097	1C 15T 1S_Cd	16
T. dobrogicus	Debrc	ZMA.RenA_9097	1C 15T 1S_Cd	16
T. dobrogicus	Debrc	ZMA.RenA_9097	1C 15T 1S_Cd	16
T. dobrogicus	Debrc	ZMA.RenA_9097	1C 16T 1S_Cd	17
T. dobrogicus	Podgorac	ZMA.RenA_9146	1C 15T 1S_Cd	16
T. dobrogicus	Podgorac	ZMA.RenA_9146	1C 16T 1S_Cd	17
T. dobrogicus	Zimnicea	ZMA.RenA_9171	1C 15T 1S_Cd	16
T. dobrogicus	Zimnicea	ZMA.RenA_9171	1C 15T 1S_Cd	16
T. dobrogicus	Tadten	ZMA.RenA_ 8041	1C 15T 1S_Cd	16
T. dobrogicus	Tadten	ZMA.RenA_ 8041	1C 15T 1S_Cd	16
T. dobrogicus	Tadten	ZMA.RenA_ 8041	1C 15T 1S_Cd	16
T. dobrogicus	Alap	ZMA.RenA_ 9083	1C 15T 1S_Cd	16
T. dobrogicus	Alap	ZMA.RenA_ 9083	1C 15T 1S_Cd	16
T. dobrogicus	Alap	ZMA.RenA_ 9083	1C 14T 1S_Cd	15
T. dobrogicus	Alap	ZMA.RenA_ 9083	1C 16T 1S_Cd	17
T. dobrogicus	Belgrade	ZMA.RenA_ 9090	1C 16T 1S_Cd	17
T. dobrogicus	Belgrade	ZMA.RenA_ 9090	1C 16T 1S_Cd	17

T. dobrogicus	Belgrade	ZMA.RenA_ 9090	1C 16T 1S_Cd	17
T. dobrogicus	Belgrade	ZMA.RenA_ 9090	1C 16T 1S_Cd	17
T. dobrogicus	Belgrade	ZMA.RenA_ 9090	1C 15T 1S_Cd	16
T. dobrogicus	Belgrade	ZMA.RenA_ 9090	1C 15T 1S_Cd	16
T. dobrogicus	Drösing	ZMA.RenA_ 9102	1C 16T 1S_Cd	17
T. dobrogicus	Drösing	ZMA.RenA_ 9102	1C 16T 1S_Cd	17
T. dobrogicus	Drösing	ZMA.RenA_ 9102	1C 16T 1S_Cd	17
T. dobrogicus	Drösing	ZMA.RenA_ 9102	1C 16T 1S_Cd	17
T. dobrogicus	Drösing	ZMA.RenA_ 9102	1C 16T 1S_Cd	17
T. dobrogicus	Ečka	ZMA.RenA_ 9104	1C 16T 1S_Cd	17
T. dobrogicus	Ečka	ZMA.RenA_ 9104	1C 16T 1S_Cd	17
T. dobrogicus	Ečka	ZMA.RenA_ 9104	1C 16T 1S_Cd	17
T. dobrogicus	Ečka	ZMA.RenA_ 9104	1C 15T 1S_Cd	16
T. dobrogicus	Ečka	ZMA.RenA_ 9104	1C 16T 1S_Cd	17
T. dobrogicus	Ečka	ZMA.RenA_ 9104	1C 16T 1S_Cd	17
T. dobrogicus	Ečka	ZMA.RenA_ 9104	1C 16T 1S_Cd	17
T. dobrogicus	Ečka	ZMA.RenA_ 9104	1C 16T 1S_Cd	17
T. dobrogicus	Ečka	ZMA.RenA_ 9104	1C 16T 1S_Cd	17
T. dobrogicus	Ečka	ZMA.RenA_ 9104	1C 16T 1S_Cd	17
T. dobrogicus	Ečka	ZMA.RenA_ 9104	1C 15T 1S_Cd	16
T. dobrogicus	Ečka	ZMA.RenA_ 9104	1C 16T 1S_Cd	17
T. dobrogicus	Ečka	ZMA.RenA_ 9104	1C 15T 0.5T/S 0.5S/Cd_Cd	16.5
T. dobrogicus	Glušci	ZMA.RenA_9111	1C 15T 1S_Cd	16
T. dobrogicus	Glušci	ZMA.RenA_9111	1C 16T 1S_Cd	17
T. dobrogicus	Glušci	ZMA.RenA_9111	1C 15T 1S_Cd	16
T. dobrogicus	Glušci	ZMA.RenA_9111	1C 16T 1S_Cd	17
T. dobrogicus	Glušci	ZMA.RenA_9111	1C 15T 1S_Cd	16
T. dobrogicus	Glušci	ZMA.RenA_9111	1C 15T 1S_Cd	16
T. dobrogicus	Glušci	ZMA.RenA_9111	1C 16T 1S_Cd	17
T. dobrogicus	Glušci	ZMA.RenA_9111	1C 15T 1S_Cd	16
T. dobrogicus	Glušci	ZMA.RenA_9111	1C 16T 1S_Cd	17
T. dobrogicus	Jamena	ZMA.RenA_9120	1C 15T 1S_Cd	16
T. dobrogicus	Jamena	ZMA.RenA_9120	1C 16T 1S_Cd	17
T. dobrogicus	Jamena	ZMA.RenA_9120	1C 15T 1S_Cd	16

T. dobrogicus	Jamena	ZMA.RenA_9120	1C 15T 1S_Cd	16
T. dobrogicus	Jamena	ZMA.RenA_9120	1C 15T 0.5T/S 0.5S/Cd_Cd	16.5
T. dobrogicus	Jamena	ZMA.RenA_9120	1C 15T 1S_Cd	16
T. dobrogicus	Jamena	ZMA.RenA_9120	1C 15T 1S_Cd	16
T. dobrogicus	Jamena	ZMA.RenA_9120	1C 15T 1S_Cd	16
T. dobrogicus	Opovo	ZMA.RenA_9142	1C 16T 1S_Cd	17
T. dobrogicus	Opovo	ZMA.RenA_9142	1C 15T 0.5T/S 0.5S/Cd_Cd	16.5
T. dobrogicus	Opovo	ZMA.RenA_9142	1C 15T 1S_Cd	16
T. dobrogicus	Opovo	ZMA.RenA_9142	1C 16T 1S_Cd	17
T. dobrogicus	Opovo	ZMA.RenA_9142	1C 16T 1S_Cd	17
T. dobrogicus	Opovo	ZMA.RenA_9142	1C 16T 1S_Cd	17
T. dobrogicus	Opovo	ZMA.RenA_9142	1C 15T 1S_Cd	16
T. dobrogicus	Senta	ZMA.RenA_9153	1C 16T 1S_Cd	17
T. dobrogicus	Senta	ZMA.RenA_9153	1C 16T 1S_Cd	17
T. dobrogicus	Senta	ZMA.RenA_9153	1C 16T 1S_Cd	17
T. dobrogicus	Senta	ZMA.RenA_9153	1C 16T 1S_Cd	17
T. dobrogicus	Senta	ZMA.RenA_9153	1C 16T 1S_Cd	17
T. dobrogicus	Senta	ZMA.RenA_9153	1C 16T 1S_Cd	17
T. dobrogicus	Senta	ZMA.RenA_9153	1C 16T 1S_Cd	17
T. dobrogicus	Senta	ZMA.RenA_9153	1C 16T 1S_Cd	17
T. dobrogicus	Senta	ZMA.RenA_9153	1C 16T 1S_Cd	17
T. dobrogicus	Senta	ZMA.RenA_9153	1C 16T 1S_Cd	17
T. dobrogicus	Senta	ZMA.RenA_9153	1C 16T 1S_Cd	17
T. dobrogicus	Senta	ZMA.RenA_9153	1C 15T 1S_Cd	16
T. dobrogicus	Senta	ZMA.RenA_9153	1C 16T 1S_Cd	17
T. dobrogicus	Senta	ZMA.RenA_9153	1C 16T 1S_Cd	17
T. ivanbureschi	Vlasi	IBISS_9834	1C 13T 1S_Cd	14
T. ivanbureschi	Vlasi	IBISS_9819	1C 12T 1S_Cd	13
T. ivanbureschi	Vlasi	IBISS_9833	1C 13T 1S_Cd	14
T. ivanbureschi	Vlasi	IBISS_9831	1C 13T 1S_Cd	14
T. ivanbureschi	Vlasi	IBISS_9824	1C 12T 1S_Cd	13
T. ivanbureschi	Vlasi	IBISS_9835	1C 13T 1S_Cd	14
T. ivanbureschi	Vlasi	IBISS_9827	1C 12T 1S_Cd	13
T. ivanbureschi	Vlasi	IBISS_9814	1C 13T 1S_Cd	14

T. ivanbureschi	Vlasi	IBISS_9825	1C 12T 1S_Cd	13
T. ivanbureschi	Vlasi	IBISS_9815	1C 12T 1S_Cd	13
T. ivanbureschi	Vlasi	IBISS_9823	1C 13T 1S_Cd	14
T. ivanbureschi	Vlasi	IBISS_9816	1C 13T 1S_Cd	14
T. ivanbureschi	Vlasi	IBISS_9822	1C 14T 0.5T/S 0.5S/Cd_Cd	15
T. ivanbureschi	Vlasi	IBISS_9828	1C 13T 1S_Cd	14
T. ivanbureschi	Vlasi	IBISS_9829	1C 12T 1S_Cd	14
T. ivanbureschi	Vlasi	IBISS_9830	1C 12T 1S_Cd	13
T. ivanbureschi	Vlasi	IBISS_9832	1C 13T 1S_Cd	14
T. ivanbureschi	Vlasi	IBISS_9818	1C 12T 0.5T/S 0.5S/Cd_Cd	13.5
T. ivanbureschi	Vlasi	IBISS_9821	1C 13T 1S_Cd	14
T. ivanbureschi	Vlasi	IBISS_9817	1C 12T 0.5T/S 0.5S/Cd_Cd	13.5
T. ivanbureschi	Vlasi	IBISS_9826	1C 12T 1S_Cd	13
T. ivanbureschi	Vlasi	IBISS_9820	1C 13T 1S_Cd	14
T. ivanbureschi	Vlasi	IBISS_G23025	1C 13T 1S_Cd	14
T. ivanbureschi	Vlasi	IBISS_G23026	1C 13T 1S_Cd	14
T. ivanbureschi	Vlasi	IBISS_G23027	1C 12T 1S_Cd	13
T. ivanbureschi	Vlasi	IBISS_G23028	1C 12T 0.5T/S 0.5S/Cd_Cd	13.5
T. ivanbureschi	Vlasi	IBISS_G23029	1C 13T 1S_Cd	14
T. ivanbureschi	Vlasi	IBISS_G23030	1C 12T 1S_Cd	13
T. ivanbureschi	Vlasi	IBISS_G23031	1C 12T 1S_Cd	13
T. ivanbureschi	Vlasi	IBISS_G23032	1C 12T 1S_Cd	13
T. ivanbureschi	Vlasi	IBISS_G23033	1C 13T 1S_Cd	14
T. ivanbureschi	Berovo	IBISS_6c33	1C 13T 1S_Cd	14
T. ivanbureschi	Berovo	IBISS_8c33	1C 12T 1S_Cd	13
T. ivanbureschi	Berovo	IBISS_4c33	1C 13T 1S_Cd	14
T. ivanbureschi	Berovo	IBISS_1c33	1C 12T 1S_Cd	13
T. ivanbureschi	Berovo	IBISS_3c33	1C 14T 0.5T/S 0.5S/Cd_Cd	15
T. ivanbureschi	Berovo	IBISS_7c33	1C 12T 1S_Cd	13
T. ivanbureschi	Berovo	IBISS_11c33	1C 12T 0.5T/S 0.5S/Cd_Cd	13.5
T. ivanbureschi	Berovo	IBISS_2c33	1C 13T 1S_Cd	14
T. ivanbureschi	Berovo	IBISS_12c33	1C 13T 1S_Cd	14
T. ivanbureschi	Berovo	IBISS_5c33	1C 13T 1S_Cd	14
T. ivanbureschi	Berovo	IBISS_9c33	1C 12T 1S_Cd	13

T. ivanbureschi	Berovo	IBISS_13c33	1C 12T 1S_Cd	13
T. ivanbureschi	Berovo	IBISS_10c33	1C 12T 1S_Cd	13
T. ivanbureschi	Berovo	IBISS_4c33	1C 13T 1S_Cd	14
T. ivanbureschi	Berovo	IBISS_9c33	1C 12T 1S_Cd	13
T. ivanbureschi	Berovo	IBISS_7c33	1C 13T 1S_Cd	14
T. ivanbureschi	Berovo	IBISS_3c33	1C 12T 1S_Cd	13
T. ivanbureschi	Berovo	IBISS_6c33	1C 12T 1S_Cd	13
T. ivanbureschi	Berovo	IBISS_1c33	1C 13T 1S_Cd	14
T. ivanbureschi	Berovo	IBISS_5c33	1C 12T 1S_Cd	14
T. ivanbureschi	Berovo	IBISS_8c33	1C 12T 0.5T/S 0.5S/Cd_Cd	13.5
T. ivanbureschi	Berovo	IBISS_2c33	1C 12T 1S_Cd	13
T. macedonicus	Rtanj	IBISS_G22953	1C 13T 1S_Cd	14
T. macedonicus	Rtanj	IBISS_G22954	1C 13T 1S_Cd	14
T. macedonicus	Rtanj	IBISS_G22955	1C 13T 1S_Cd	14
T. macedonicus	Rtanj	IBISS_G22956	1C 13T 1S_Cd	14
T. macedonicus	Rtanj	IBISS_G22957	1C 13T 1S_Cd	14
T. macedonicus	Rtanj	IBISS_G22958	1C 12T 1S_Cd	13
T. macedonicus	Rtanj	IBISS_G22959	1C 13T 1S_Cd	14
T. macedonicus	Rtanj	IBISS_G22960	1C 12T 1S_Cd	13
T. macedonicus	Rtanj	IBISS_G22961	1C 13T 1S_Cd	14
T. macedonicus	Rtanj	IBISS_G22962	1C 12T 1S_Cd	13
T. macedonicus	Rtanj	IBISS_G22970	1C 12T 0.5T/S 0.5S/Cd_Cd	13.5
T. macedonicus	Rtanj	IBISS_G22971	1C 13T 1S_Cd	14
T. macedonicus	Rtanj	IBISS_G22969	1C 12T 1S_Cd	13
T. macedonicus	Rtanj	IBISS_G22972	1C 12T 1S_Cd	13
T. macedonicus	Rtanj	IBISS_G22964	1C 13T 0.5T/S 0.5S/Cd_Cd	14.5
T. macedonicus	Rtanj	IBISS_G22963	1C 14T 1S_Cd	15
T. macedonicus	Rtanj	IBISS_G22965	1C 13T 1S_Cd	14
T. macedonicus	Rtanj	IBISS_G22966	1C 13T 1S_Cd	14
T. macedonicus	Rtanj	IBISS_G22968	1C 12T 1S_Cd	13
T. macedonicus	Rtanj	IBISS_G22967	1C 13T 1S_Cd	14
T. macedonicus	Vranje-Sveti Ilija	IBISS_џ6220	1C 13T 1S_Cd	14
T. macedonicus	Vranje-Sveti Ilija	IBISS_џ6216	1C 13T 1S_Cd	14
T. macedonicus	Vranje-Sveti Ilija	IBISS_џ6215	1C 13T 1S_Cd	14

T. macedonicus	Vranje-Sveti Ilija	IBISS_14c7	1C 13T 1S_Cd	14
T. macedonicus	Vranje-Sveti Ilija	IBISS_џ6224	1C 13T 1S_Cd	14
T. macedonicus	Vranje-Sveti Ilija	IBISS_џ6222	1C 14T 1S_Cd	15
T. macedonicus	Vranje-Sveti Ilija	IBISS_џ6226	1C 13T 1S_Cd	14
T. macedonicus	Vranje-Sveti Ilija	IBISS_џ6221	1C 13T 1S_Cd	14
T. macedonicus	Vranje-Sveti Ilija	IBISS_џ6214	1C 13T 1S_Cd	14
T. macedonicus	Vranje-Sveti Ilija	IBISS_џ6228	1C 13T 1S_Cd	14
T. macedonicus	Vranje-Sveti Ilija	IBISS_џ6230	1C 13T 0.5T/S 0.5S/Cd_Cd	14.5
T. macedonicus	Vranje-Sveti Ilija	IBISS_џ6213	1C 13T 1S_Cd	14
T. macedonicus	Vranje-Sveti Ilija	IBISS_џ6223	1C 13T 1S_Cd	14
T. macedonicus	Vranje-Sveti Ilija	IBISS_џ6232	1C 12T 1S_Cd	13
T. macedonicus	Vranje-Sveti Ilija	IBISS_11c7	1C 12T 1S_Cd	13
T. macedonicus	Vranje-Sveti Ilija	IBISS_11c7	1C 13T 1S_Cd	14
T. macedonicus	Vranje-Sveti Ilija	IBISS_21c7	1C 13T 1S_Cd	14
T. macedonicus	Vranje-Sveti Ilija	IBISS_18c7	1C 13T 1S_Cd	14
T. macedonicus	Vranje-Sveti Ilija	IBISS_17c7	1C 12T 1S_Cd	13
T. cristatus × *T. marmoratus*	Mayenne	ZMA.RenA_7364	1C 14T 1S_Cd	15
T. cristatus × *T. marmoratus*	Mayenne	ZMA.RenA_7419	1C 13T 1S_Cd	14
T. cristatus × *T. marmoratus*	Mayenne	ZMA.RenA_7525	1C 14T 1S_Cd	15
T. cristatus × *T. marmoratus*	Mayenne	ZMA.RenA_7526	1C 13T 1S_Cd	14
T. cristatus × *T. marmoratus*	Mayenne	ZMA.RenA_7527	1C 13T 1S_Cd	14
T. cristatus × *T. marmoratus*	Mayenne	ZMA.RenA_7528	1C 12T 1S_Cd	13
T. cristatus × *T. marmoratus*	Mayenne	ZMA.RenA_7529	1C 12T 1S_Cd	13
T. cristatus × *T. marmoratus*	Mayenne	ZMA.RenA_7531	1C 13T 1S_Cd	14
T. cristatus × *T. marmoratus*	Mayenne	ZMA.RenA_7532	1C 12T 1S_Cd	13
T. cristatus × *T. marmoratus*	Mayenne	ZMA.RenA_7533	1C 12T 1S_Cd	13
T. cristatus × *T. marmoratus*	Mayenne	ZMA.RenA_7534	1C 12T 1S_Cd	13
T. cristatus × *T. marmoratus*	Mayenne	ZMA.RenA_7535	1C 13T 1S_Cd	14
T. cristatus × *T. marmoratus*	Mayenne	ZMA.RenA_7536	1C 12T 1S_Cd	13
T. cristatus × *T. marmoratus*	Mayenne	ZMA.RenA_7537	1C 12T 1S_Cd	13
T. cristatus × *T. marmoratus*	Mayenne	ZMA.RenA_7538	1C 13T 1S_Cd	14
T. cristatus × *T. marmoratus*	Mayenne	ZMA.RenA_7539	1C 12T 1S_Cd	13
T. cristatus × *T. marmoratus*	Mayenne	ZMA.RenA_7541	1C 11T 1S_Cd	12
T. cristatus × *T. marmoratus*	Mayenne	ZMA.RenA_7542	1C 12T 1S_Cd	13

T. cristatus × *T. marmoratus*	Mayenne	ZMA.RenA_7543	1C 12T 1S_Cd	13
T. cristatus × *T. marmoratus*	Mayenne	ZMA.RenA_7544	1C 12T 1S_Cd	13
T. cristatus × *T. marmoratus*	Mayenne	ZMA.RenA_7545	1C 13T 1S_Cd	14
T. cristatus × *T. marmoratus*	Mayenne	ZMA.RenA_7598	1C 12T 1S_Cd	13
T. cristatus × *T. marmoratus*	Mayenne	ZMA.RenA_7607	1C 12T 1S_Cd	13
T. cristatus × *T. marmoratus*	Mayenne	ZMA.RenA_7608	1C 12T 1S_Cd	13
T. cristatus × *T. marmoratus*	Mayenne	ZMA.RenA_7609	1C 14T 1S_Cd	15
T. cristatus × *T. marmoratus*	Mayenne	ZMA.RenA_7610	1C 12T 1S_Cd	13
T. cristatus × *T. marmoratus*	Mayenne	ZMA.RenA_7611	1C 12T 1S_Cd	13
T. cristatus × *T. marmoratus*	Mayenne	ZMA.RenA_7612	1C 12T 1S_Cd	13
T. cristatus × *T. marmoratus*	Mayenne	ZMA.RenA_7620	1C 12T 1S_Cd	13
T. cristatus × *T. marmoratus*	Mayenne	ZMA.RenA_7621	1C 12T 1S_Cd	13
T. cristatus × *T. marmoratus*	Mayenne	ZMA.RenA_7624	1C 12T 1S_Cd	13
T. cristatus × *T. marmoratus*	Mayenne	ZMA.RenA_7625	1C 13T 1S_Cd	14
T. cristatus × *T. marmoratus*	Mayenne	ZMA.RenA_7864	1C 11T 0.5T/S 0.5S/Cd_Cd	12.5
T. cristatus × *T. marmoratus*	Mayenne	ZMA.RenA_8033	1C 11T 1S_Cd	12
T. cristatus × *T. marmoratus*	Mayenne	ZMA.RenA_8033	1C 13T 1S_Cd	14
T. cristatus × *T. marmoratus*	Mayenne	ZMA.RenA_8033	1C 12T 1S_Cd	13
T. cristatus × *T. marmoratus*	Mayenne	ZMA.RenA_8052	1C 12T 1S_Cd	13
T. cristatus × *T. marmoratus*	Mayenne	ZMA.RenA_8057	1C 12T 1S_Cd	13
T. cristatus × *T. marmoratus*	Mayenne	ZMA.RenA_8058	1C 14T 1S_Cd	15
T. cristatus × *T. marmoratus*	Mayenne	ZMA.RenA_8058	1C 14T 1S_Cd	15
T. cristatus × *T. marmoratus*	Mayenne	ZMA.RenA_8058	1C 14T 1S_Cd	15
T. cristatus × *T. marmoratus*	Mayenne	ZMA.RenA_8058	1C 13T 1S_Cd	14
T. cristatus × *T. marmoratus*	Mayenne	ZMA.RenA_8058	1C 12T 0.5T/S 0.5S/Cd_Cd	13.5
T. cristatus × *T. marmoratus*	Mayenne	ZMA.RenA_8058	1C 12T 1S_Cd	13
T. cristatus × *T. marmoratus*	Mayenne	ZMA.RenA_8058	1C 12T 1S_Cd	13
T. cristatus × *T. marmoratus*	Mayenne	ZMA.RenA_9058	1C 12T 1S_Cd	13
T. cristatus × *T. marmoratus*	Mayenne	ZMA.RenA_9058	1C 13T 1S_Cd	14
T. cristatus × *T. marmoratus*	Mayenne	ZMA.RenA_9058	1C 13T 1S_Cd	14
T. cristatus × *T. marmoratus*	Mayenne	ZMA.RenA_9059	1C 12T 1S_Cd	13
T. cristatus × *T. marmoratus*	Mayenne	ZMA.RenA_9060	1C 12T 1S_Cd	13
T. cristatus × *T. marmoratus*	Mayenne	ZMA.RenA_9060	1C 12T 1S_Cd	13
T. cristatus × *T. marmoratus*	Mayenne	ZMA.RenA_9060	1C 12T 0.5T/S 0.5S/Cd_Cd	13.5

T. cristatus × *T. marmoratus*	Mayenne	ZMA.RenA_9061	1C 12T 1S_Cd	13
T. cristatus × *T. marmoratus*	Mayenne	ZMA.RenA_9062	1C 12T 1S_Cd	13
T. cristatus × *T. marmoratus*	Mayenne	ZMA.RenA_9062	1C 12T 1S_Cd	13
T. cristatus × *T. marmoratus*	Mayenne	ZMA.RenA_9063	1C 13T 1S_Cd	14
T. cristatus × *T. marmoratus*	Mayenne	ZMA.RenA_9064	1C 12T 0.5T/S 0.5S/Cd_Cd	13.5
T. cristatus × *T. marmoratus*	Mayenne	ZMA.RenA_9065	1C 13T 1S_Cd	14
T. cristatus × *T. marmoratus*	Mayenne	ZMA.RenA_9066	1C 12T 1S_Cd	13
T. cristatus × *T. marmoratus*	Mayenne	ZMA.RenA_9067	1C 12T 1S_Cd	13
T. cristatus × *T. marmoratus*	Mayenne	ZMA.RenA_9067	1C 12T 1S_Cd	13
T. cristatus × *T. marmoratus*	Mayenne	ZMA.RenA_9067	1C 12T 1S_Cd	13
T. cristatus × *T. marmoratus*	Jublians	ZMA.RenA_9243	1C 12T 1S_Cd	13
T. cristatus × *T. marmoratus*	Jublians	ZMA.RenA_9243	1C 12T 1S_Cd	13
T. cristatus × *T. marmoratus*	Mayenne	ZMA.RenA_9322	1C 12T 1S_Cd	13
T. cristatus × *T. marmoratus*	Mayenne	ZMA.RenA_9241	1C 13T 1S_Cd	14
T. cristatus × *T. marmoratus*	Mayenne	ZMA.RenA_9241	1C 13T 1S_Cd	14
T. cristatus × *T. marmoratus*	Mayenne	ZMA.RenA_9241	1C 12T 0.5T/S 0.5S/Cd_Cd	13.5
